# A consensus and saturated genetic map provides insight into genome anchoring, synteny of Solanaceae and leaf- and fruit-related QTLs in wolfberry (*Lycium* Linn.)

**DOI:** 10.1186/s12870-021-03115-1

**Published:** 2021-07-24

**Authors:** Jianhua Zhao, Haoxia Li, Yuhui Xu, Yue Yin, Ting Huang, Bo Zhang, Yajun Wang, Yanlong Li, Youlong Cao, Wei An

**Affiliations:** 1 Wolfberry Science Research Institute, Ningxia Academy of Agriculture and Forestry Sciences/National Wolfberry Engineering Research Center, Yinchuan, 750002 China; 2 Desertification Control Research Institute, Ningxia Academy of Agriculture and Forestry Sciences, Yinchuan, 750002 China; 3 Adsen Biotechnology Co., Ltd, Urumchi, 830022 China

**Keywords:** Genetic map, Resequencing, Leaf- and fruit-related traits, QTL, Genome synteny, *Lycium* Linn

## Abstract

**Background:**

*Lycium* Linn. (Solanaceae) is a genus of economically important plants producing fruits and leaves with high nutritional value and medicinal benefits. However, genetic analysis of this plant and molecular breeding for quality improvement are limited by the lack of sufficient molecular markers.

**Results:**

In this study, two parental strains, ‘Ningqi No. 1’ (*Lycium barbarum* L.) and ‘Yunnan Gouqi’ (*Lycium yunnanense* Kuang et A.M. Lu), and 200 F_1_ hybrid individuals were resequenced for genetic analysis. In total, 8,507 well-selected SNPs were developed, and a high-density genetic map (NY map) was constructed with a total genetic distance of 2,122.24 cM. A consensus genetic map was established by integrating the NY map and a previously published genetic map (NC map) containing 15,240 SNPs, with a total genetic distance of 3,058.19 cM and an average map distance of 0.21 cM. The 12 pseudochromosomes of the *Lycium* reference genome were anchored using this consensus genetic map, with an anchoring rate of 64.3%. Moreover, weak collinearities between the consensus map and the pepper, potato, and tomato genomes were observed. Twenty-five stable QTLs were identified for leaf- and fruit-related phenotypes, including fruit weight, fruit longitude, leaf length, the fruit index, and the leaf index; these stable QTLs were mapped to four different linkage groups, with LOD scores ranging from 2.51 to 19.37 and amounts of phenotypic variance explained from 6.2% to 51.9%. Finally, 82 out of 188 predicted genes underlying stable QTLs for fruit-related traits were differentially expressed according to RNA-seq analysis.

**Conclusions:**

A chromosome-level assembly can provide a foundation for further functional genomics research for wolfberry. The genomic regions of these stably expressed QTLs could be used as targets for further fine mapping and development of molecular markers for marker-assisted selection (MAS). The present study provided valuable information on saturated SNP markers and reliable QTLs for map-based cloning of functional genes related to yield and morphological traits in *Lycium* spp.

**Supplementary Information:**

The online version contains supplementary material available at 10.1186/s12870-021-03115-1.

## Background

*Lycium* Linn. (Solanaceae) is a genus of perennial shrubs with > 80 species worldwide and is mainly distributed in South America, southwestern North America, southern Africa, and eastern Asia [1]. Seven species and three varieties of *Lycium* occur in China [2]; of these, *Lycium barbarum* (‘goji berry’ or Chinese wolfberry) has been domesticated and widely cultivated in Northwest China for > 600 years [2, 3]. The edible fruits and leaves of *L. barbarum* are used as functional foods and traditional Chinese medicinal herbs in China [4, 5]. Many compounds from *L. barbarum* fruits and leaves, including flavonoids, carotenoids, and polysaccharides, have been reported to be closely associated with the health-enhancing effects of this species [5, 6]. However, it is difficult to improve the quality of *Lycium* because of the unclear molecular genetic mechanisms underlying *Lycium* fruit and leaf traits.

Next-generation sequencing (NGS) coupled with the growing number of reference genome sequences presents an opportunity to redesign genotyping strategies for more effective genetic mapping and genome analysis [7], which can result in ultra-high-density linkage map construction and localized quantitative trait loci (QTLs) for multiple traits [8, 9]. Resequencing and high-density genetic mapping in crops with complete genome sequences identified *days to heading8* (*Dth8*) and *lax panicle1* (*Lax1*) as candidate genes in rice [10] and sequence alterations in a novel ion transporter gene (*GmCHX1*) inducing salt tolerance in wild soybean [11]. Moreover, structural variations were reported in allotetraploid cotton [12]. With the decreasing costs of sequencing technologies, whole-genome sequencing has been applied to an increasing number of plant species; in addition, the numerous single nucleotide polymorphism (SNP) markers developed by aligning resequencing data to the corresponding reference genome can provide a powerful approach for deciphering the genetic basis of complex traits and for large-scale gene discovery [13].

**Fig. 1:**
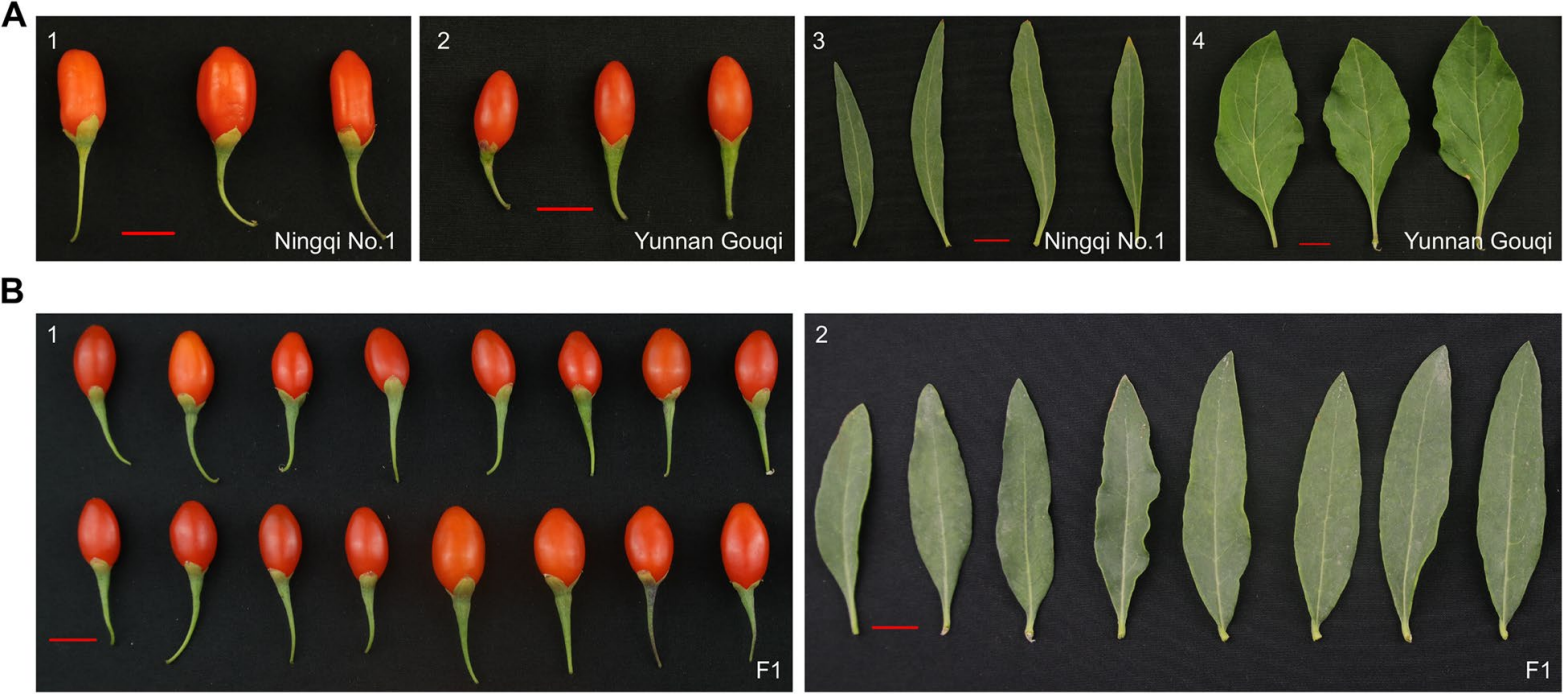
Appearance of fruits and leaves of the two parents (A) and representative F_1_ individuals (B) of *Lycium*. Note: **A**-1 female parent ‘Ningqi No. 1’ fruits; **A**-2 male parent ‘Yunnan Gouqi’ fruits; **A**-3 female parent ‘Ningqi No. 1’ leaves; **A**-4 male parent ‘Yunnan Gouqi’ leaves; **B** a selection of the hybrid offspring showing differences in fruits and leaves

The first sequencing-based linkage map for *Lycium* was constructed by specific length amplified fragment sequencing (SLAF-seq) using a diploid F_1_ population derived from a cross between ‘Ningqi No. 1’ (NN) and ‘Chinese gouqi’, and 18 stable leaf and fruit QTLs were mapped onto the resulting genetic map [14]. Recently, a 1,891-Mb *Lycium* genome sequence (Cao et al., unpublished 2021) provided an opportunity to develop SNP markers for population genotyping. In the present study, we used an F_1_ population (Fig. 1) of *Lycium* with the shared parent ‘Ningqi No. 1’ to map QTLs for agronomic traits. Genotyping was performed using resequencing followed by SNP identification. The resulting SNPs were used to construct a high-density linkage map and an integrated consensus map. Using these maps, we were able to map yield-related QTLs in *Lycium*. Such QTLs and closely linked markers could then be used for molecular breeding to improve *Lycium* yield and quality.

## Results

### Leaf and fruit trait variation

**Fig. 2:**
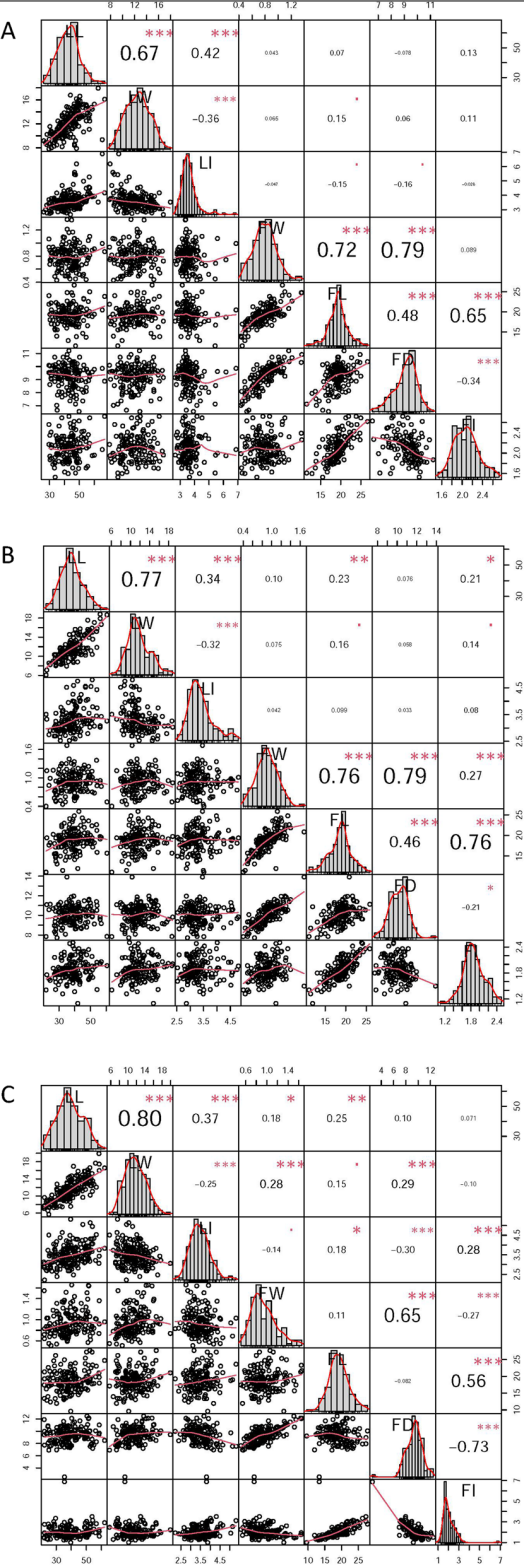
The variation and Pearson pairwise correlation analyses of leaf-related and fruit-related traits of the F_1_ population. (**A**), (**B**) and (**C**) represent the variation and Pearson pairwise correlations in 2016, 2017 and 2018, respectively. The correlations were calculated with Spearman correlation coefficients, and the *P* values are indicated as follows: *, *P* < 0.05; **, *P* < 0.01; ***, *P* < 0.001. The analysis was performed using the R package PerformanceAnalytics. Histograms for LL (leaf length), LW (leaf width), LI (leaf index), FW (fruit weight), FL (fruit length), FD (fruit diameter) and FI (fruit index) are displayed along the diagonal

Seven leaf- and fruit-related phenotypic traits were measured for three continuous years from 2016 to 2018. The coefficient of variation of most phenotypic traits was > 10%, with the highest value for fruit index (FI) in 2018 (30%) and the lowest (9%) for fruit diameter (FD) in 2016 (Table 1), indicating that all traits showed natural variation in the F_1_ population. The seven traits were normally or partially normally distributed (Table 1 and Fig. 2A-C). Correlation analysis revealed significant or extremely significant positive correlations between leaf width (LW) and leaf length (LL), leaf index (LI) and LL, fruit longitude (FL) and LL, FI and LL, and LI and FI (*P* < 0.05) and between single-fruit weight (FW) and FL, FW and FD, and FI and FL (*P* < 0.01), respectively, whereas a significant or extremely negative correlation was observed between pairs LI and LW and LI and FD (*P* < 0.05) and between FI and FD (*P* < 0.01), respectively (Fig. 2A-C).

**Table 1:** Data summary for *Lycium* phenotypes

Trait	Year	Mean ± SD	Maximum	Minimum	Skewness	Kurtosis	Coefficient of variation (%)	Variance	Shapiro–Wilk *P* value
Leaf length	2016	45.46 ± 7.69	73.13	26.26	0.48	0.67	17.00	59.19	0.010
	2017	38.84 ± 7.71	60.49	21.79	0.30	-0.21	20.00	59.39	0.278
	2018	39.33 ± 9.03	62.16	21.73	0.15	-0.67	23.00	81.47	0.042
Leaf width	2016	12.12 ± 2.09	17.95	7.81	0.10	-0.62	17.00	4.39	0.153
	2017	11.78 ± 2.38	18.81	6.10	0.59	0.17	20.00	5.68	0.003
	2018	11.41 ± 2.54	19.92	5.70	0.36	-0.14	22.00	6.47	0.097
Leaf index	2016	3.60 ± 0.58	6.88	2.66	2.14	7.65	16.00	0.33	0.000
	2017	3.32 ± 0.48	4.80	2.53	1.05	0.86	14.00	0.23	0.000
	2018	3.50 ± 0.52	5.32	2.21	0.58	0.74	15.00	0.27	0.007
Fruit weight	2016	0.79 ± 0.18	1.36	0.43	0.34	0.27	23.00	0.03	0.146
	2017	0.91 ± 0.22	1.68	0.40	0.20	0.15	25.00	0.05	0.436
	2018	0.95 ± 0.24	1.66	0.51	0.76	0.30	12.00	0.06	0.000
Fruit longitude	2016	19.25 ± 2.40	26.67	11.55	0.00	1.03	14.00	5.75	0.032
	2017	18.68 ± 2.63	25.85	11.06	-0.19	0.38	17.00	6.89	0.119
	2018	18.67 ± 3.23	27.74	9.60	0.33	0.24	10.00	10.46	0.176
Fruit diameter	2016	9.22 ± 0.89	11.22	6.55	-0.62	0.20	9.00	0.80	0.003
	2017	10.08 ± 0.95	13.91	7.72	0.13	0.79	14.00	0.90	0.053
	2018	9.39 ± 1.29	12.47	2.55	-0.82	3.82	12.00	1.66	0.000
Fruit index	2016	2.10 ± 0.25	2.72	1.53	0.34	-0.19	13.00	0.06	0.110
	2017	1.87 ± 0.24	2.50	1.10	0.13	0.19	13.00	0.06	0.249
	2018	2.05 ± 0.62	6.88	1.00	3.30	21.75	30.00	0.38	0.000

### Variation calling and annotation

**Fig. 3:**
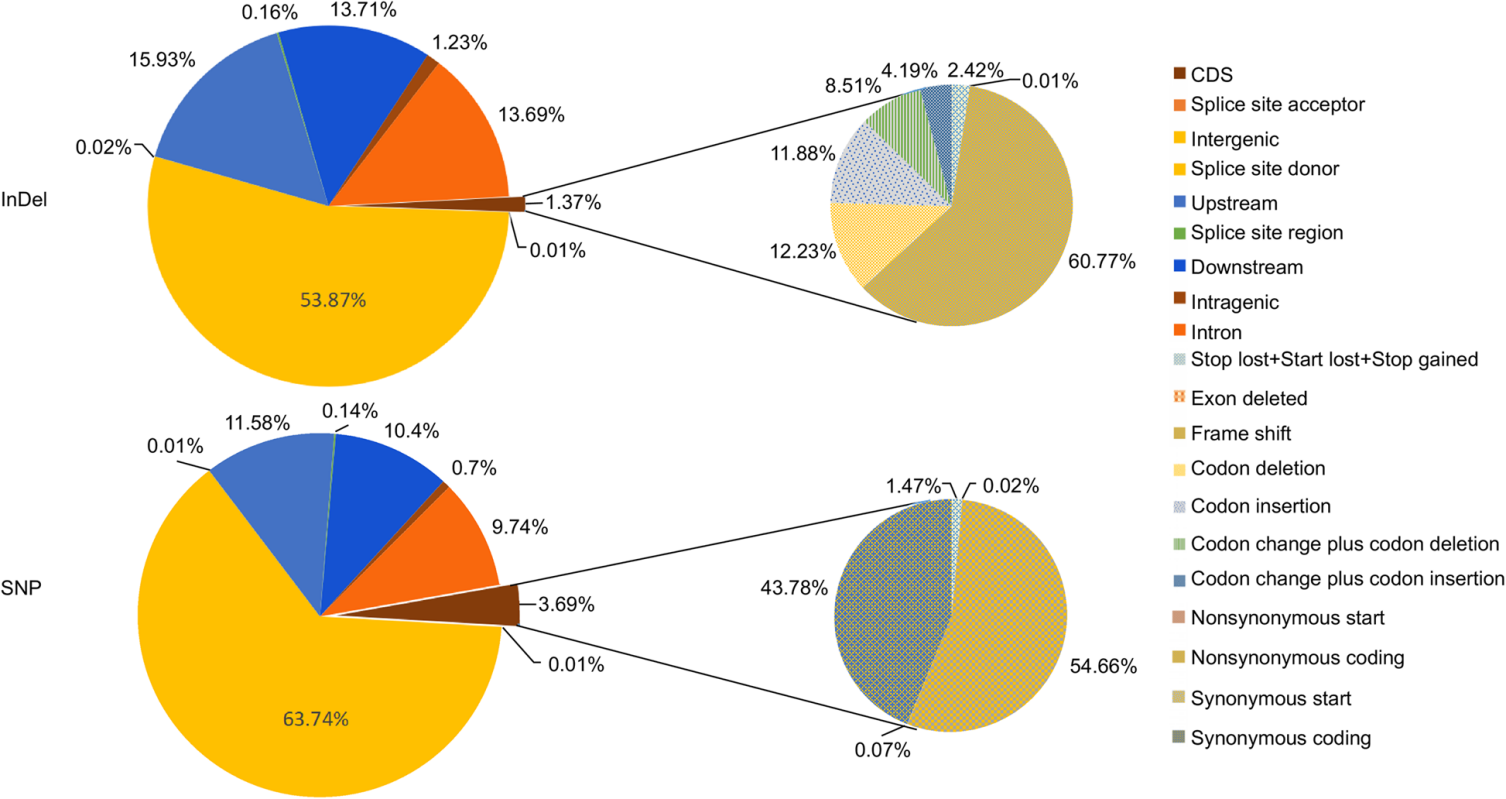
Genome variations and annotations. **A** Circos plot of SNP and InDel distributions. The outer ring indicates the SNP distribution, whereas the inner ring indicates the InDel distribution; **B** Pie charts of InDel annotations; **C** Pie charts of SNP annotation information

Whole-genome resequencing generated a total of 4,549 million clean paired-end reads, 23,968 and 227,408 million in the female and male parents, respectively, with a Q30 value of 92.06%, indicating that high-quality source data were generated. The average depths in the female and male parents were 34 × and 18 × , respectively, and the average depth in the offspring was 2.58 × (Supplementary Table 1). All clean reads were mapped onto the scaffolds of the *Lycium* reference genome, with 9,015,078 SNPs and 1,317,594 InDels called between the parents. The variation maps of SNPs and InDels are shown in Fig. 3. The SNP density was 4,880 per Mb, and the InDel density was 714 per Mb. Most annotated SNPs (63.74%) were located in intergenic regions, whereas in the coding sequence (CDS) region, most SNPs were nonsynonymous (54.66%). Similar to the SNPs, more than half of the InDels (53.87%) were annotated in intergenic regions, whereas 1.37% were located in the CDS. Of the CDS InDels, 60.77% gave rise to frameshift mutations (Fig. 3). Of all the SNPs, 8,734,495 were successfully classified into eight genotyping patterns, and a set of 3,451,010 SNPs (excluding those with pattern type aa × bb) were used to construct a high-density genetic map of *Lycium*.

### Construction of an ultradense genetic map and a consensus genetic map

To guarantee a set of high-quality SNP markers, SNPs with a depth in the parents < 10 × , SNPs with an integrity ≤ 70%, and highly significant SNPs with SD (*P* < 0.01) were filtered out. Finally, a set of 10,446 SNPs was used to construct a high-density genetic map of wolfberry, into which 8,507 SNPs were successfully integrated (Supplementary Fig. 1A). The integrated genetic map included 12 linkage groups (LGs) with a total genetic distance of 2,122.24 cM and an average map distance of 0.25 cM. LG07 included the largest number of markers (1,035), with an average distance of 0.18 cM and a total genetic distance of 182.59 cM. The smallest number of markers (510) was in LG03, spanning 119.69 cM with an average distance of 0.24 cM. The largest gap stretched across 19.41 cM in LG11. The ratios of genetic distance between adjacent markers < 5 cM ranged from 98.19% to 99.83% in LG05 and LG02, respectively (Table 2). To further integrate our published map [14], a consensus genetic map was constructed, which contained 15,240 SNPs with a total genetic distance of 3,058.19 cM and an average map distance of 0.21 cM (Table 2 and Supplementary Fig. 1B). Compared with the NY map, this consensus map harbored 6,733 more SNPs and 0.04 cM less average distance, indicating a higher resolution and likely the genetic map with the largest number of SNP markers for ligneous plants. In addition, the maximum gap was also somewhat narrowed. The consensus map represented a comprehensive improvement.

**Table 2:** Summary of the total number of SNP markers in the 12 linkage groups

Linkage group	Total markers		Total distance (cM)		Average distance (cM)		Maximum gap (cM)		Gaps < 5 cM (%)	
	NY genetic map	Consensus map	NY genetic map	Consensus map	NY genetic map	Consensus map	NY genetic map	Consensus map	NY genetic map	Consensus map
LG01	735	921	218.65	278.29	0.3	0.30	15.4	6.02	99.73	99.78
LG02	576	1262	158.03	270.61	0.27	0.21	8.41	8.41	99.83	99.92
LG03	510	1197	119.69	183.01	0.24	0.15	8.56	11.70	98.82	99.58
LG04	518	1097	159.09	229.71	0.31	0.21	11.79	8.49	99.23	99.73
LG05	608	812	193.19	261.22	0.32	0.32	17.86	17.86	98.19	98.77
LG06	766	1436	231.03	328.13	0.3	0.23	12.16	12.16	98.95	99.37
LG07	1,035	1725	182.59	267.50	0.18	0.16	13.7	13.70	99.61	99.65
LG08	752	1212	232.82	324.95	0.31	0.27	9.49	9.49	99.33	99.34
LG09	819	1437	190.72	250.42	0.23	0.17	9.57	9.57	99.63	99.72
LG10	901	1395	188.58	268.11	0.21	0.19	13.07	13.07	98.89	99.5
LG11	569	1518	165.38	248.61	0.29	0.16	19.41	19.41	98.94	99.67
LG12	718	1228	82.47	147.63	0.12	0.12	14.61	6.06	99.16	99.84
Total	8,507	15,240	2,122.24	3058.19	0.25	0.21	19.41	11.33	99.19	99.57

### Genetic map-assisted genome assembly

**Fig. 4:**
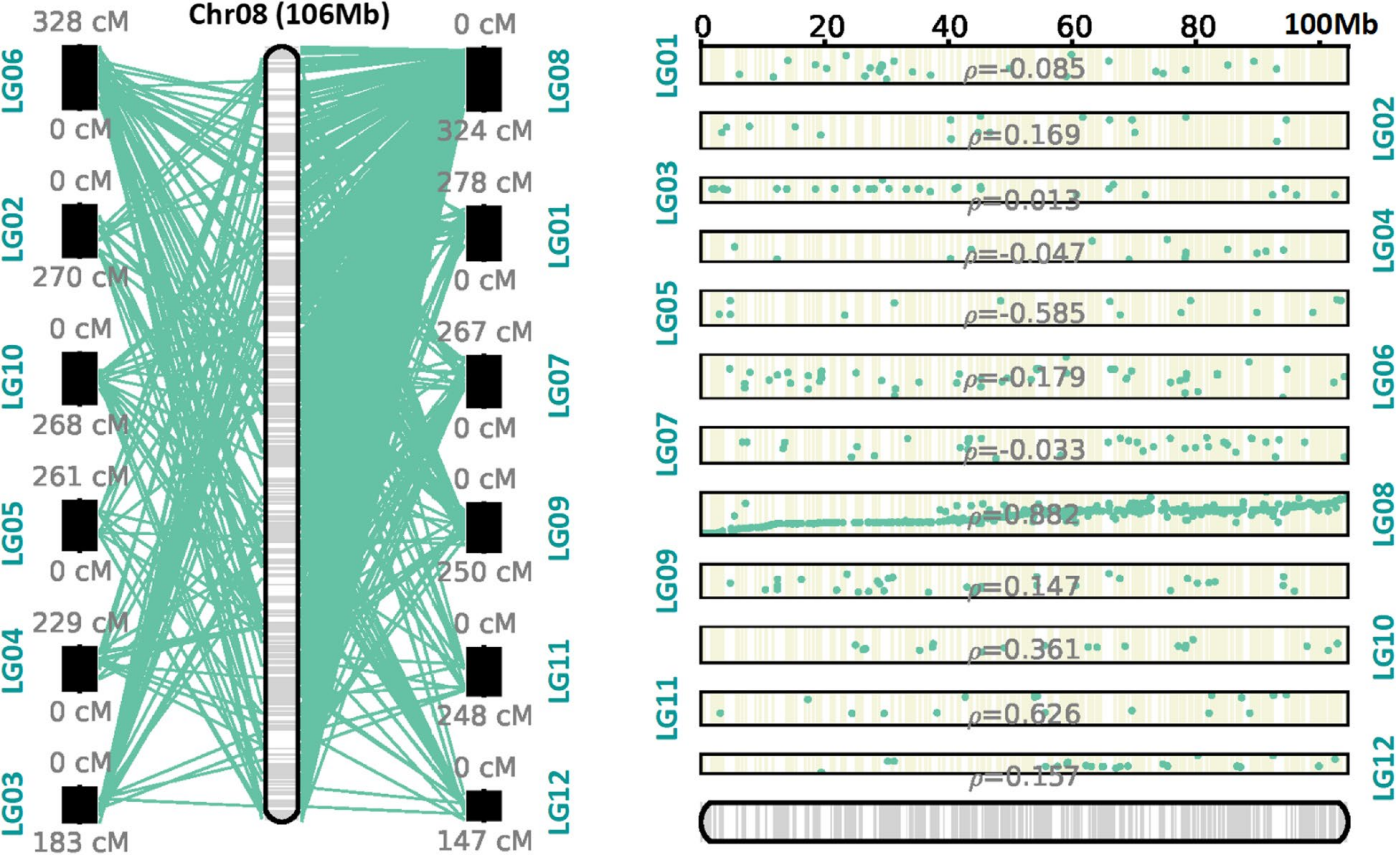
Pseudochrome 8 of *Lycium* anchored by the consensus map. The left panel represents synteny between the consensus map and the reference genome. The right panel represents the correlations between the consensus map and the reference genome. The ρ-values represent the Pearson correlation coefficients

High-density linkage maps can assist in chromosome-level genome assembly. To assemble the genome of *Lycium* at the chromosome level on the basis of our high-density consensus genetic map, we used ALLMAPS. Finally, ~ 1.21 Gb of scaffolds were mounted to 12 pseudochromosomes of *Lycium*, accounting for ~ 64.3% of the genome sequence, 51.3% of which were oriented (Fig. 4; Supplementary Fig. 2; Table 3). The longest pseudochromosome was LG10, with a total length of 132.33 Mb, whereas only 57 Mb and 57.69 Mb were mounted onto LG05 and LG01, respectively, in line with the trends in SNP numbers in the genetic map. More scaffolds were not mounted (783,254), among which scaffolds < 1 kb accounted for 99.3% (777,737/783,254). ALLMAPS scaffolding was performed by inferring and maximizing the collinearity between the genetic map and scaffolds/contigs. By comparing the collinearities between all LGs and pseudochromosomes, we found that certain collinearities occurred between each LG and the corresponding pseudochromosomes. Pseudochromosome 04 showed the lowest *P* value. Moreover, the *P* values of collinear pseudochromosomes 01, 02, 08, 09, and 11 were all > 0.8 (Supplementary Fig. 2).

**Table 3:** High-density genetic map-assisted genome assembly

Index	Anchored	Oriented	Unplaced
Unique mapped markers	12,442	10,540	2,162
Markers per Mb	10.3	10.9	3.2
Scaffolds	1,643	1,044	783,254
Scaffolds with 1 anchored marker	336	0	104
Scaffolds with 2 anchored markers	111	77	187
Scaffolds with 3 anchored markers	167	110	70
Scaffolds with ≥ 4 anchored markers	1,029	857	181
Total bases (bp)	1,208,281,949	963,551,338	670,115,130
Mapping rate (%)	64.3	51.3	35.7

### Synteny analyses

**Fig. 5:**
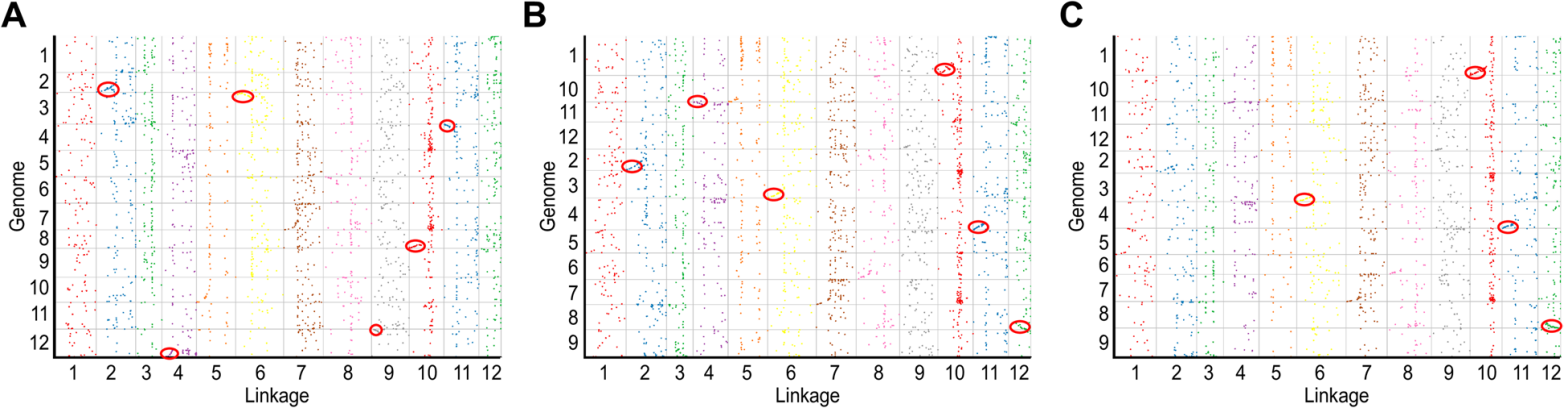
Synteny analyses between the genetic map of *Lycium* and the genomes of pepper (**A**), potato (**B**), and tomato (**C**)

We used the consensus genetic map to perform collinearity analysis with the reference genomes of pepper, potato, and tomato. Weak collinearity was found between wolfberry and the three Solanaceae species, and most regions located at the ends of chromosomes showed high collinearity. The collinear segment pairs between wolfberry and pepper were LG02-chromosome 02, LG04-chromosome 12, LG06-chromosome 03, LG09-chromosome 12, LG10-chromosome 08, and LG11-chromosome 04. The collinear segment pairs of wolfberry and potato were LG02-chromosome 02, LG06-chromosome 03, LG04-chromosome 11, G10-chromosome 01, LG11-chromosome 04, and LG12-chromosome 08. The collinear segment pairs of wolfberry and tomato were LG06-chromosome 03, LG10-chromosome 01, LG11-chromosome 04, and LG12-chromosome 08 (Fig. 5). Among these collinearity pairs, there were overlaps between wolfberry-pepper pairs and wolfberry-tomato pairs on chromosomes 02, 04, 06, 10, and 11 of wolfberry, whereas overlaps between wolfberry-tomato pairs and wolfberry-potato pairs were observed on chromosomes 06, 10, 11, and 12 of wolfberry. Furthermore, there was a potential chromosomal inversion between wolfberry-tomato pairs and wolfberry-potato pairs on chromosome 10 of wolfberry.

### QTL mapping

Using the resequencing genetic map and continuous phenotypic data from 2016 to 2018, a large number of QTLs responsible for seven agronomic traits were mapped. The QTLs were distributed in all the LGs of wolfberry except LG08, with phenotypic variance explained (PVE) values ranging from 6% to 73.6%, and the maximum LOD value was 21.39. The fruit-related QTLs were mapped mainly to LG06, LG07, LG09, LG10, and LG12, whereas the leaf-related QTLs were located mainly in LG01, LG06, LG09, and LG10 (Supplementary Table 2).

**Fig. 6:**
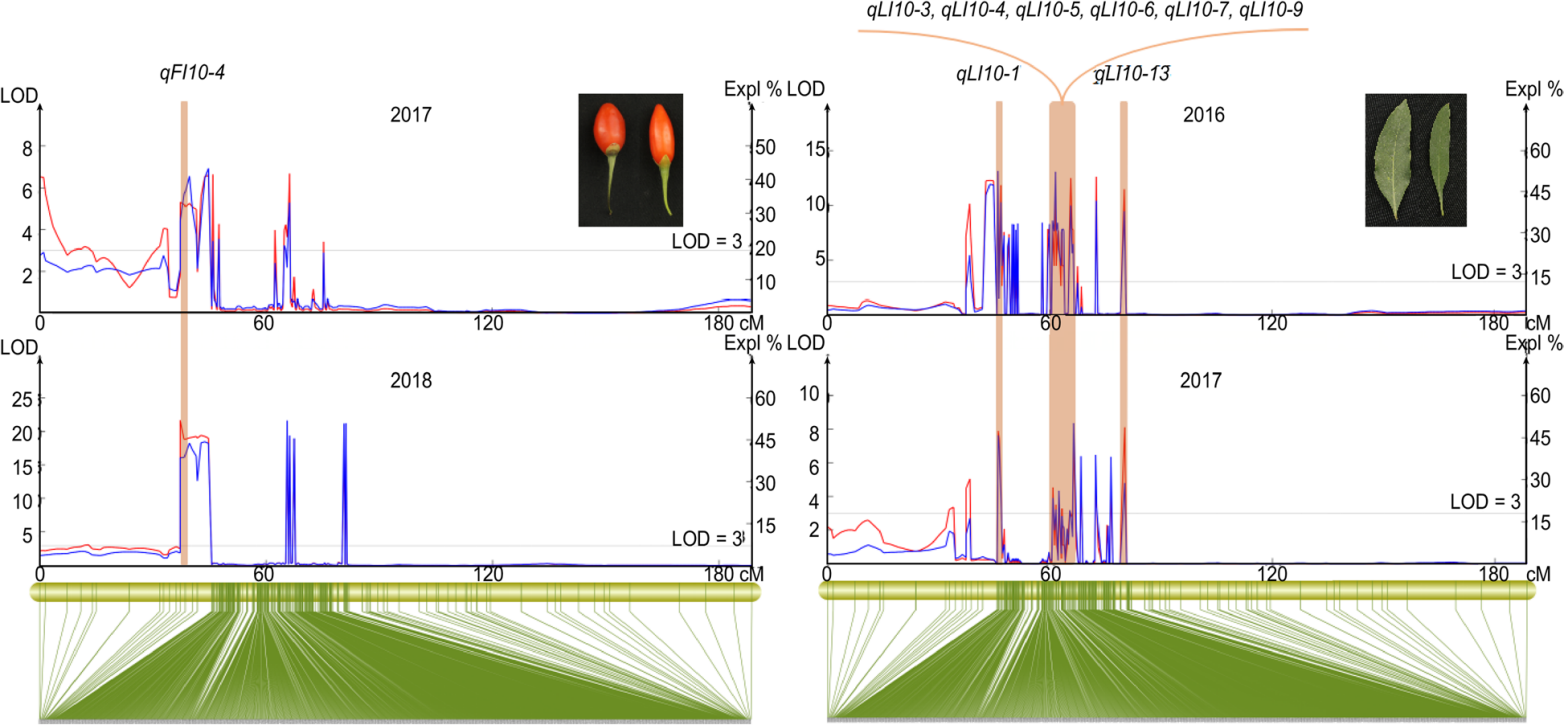
Stable QTLs for the fruit index (2017, 2018) and leaf index (2016, 2017) with higher confidence intervals on chromosome 10

We further screened stable QTLs detected in at least two years, and a total of 25 such QTLs were identified (Supplementary Table 3). We identified 13 QTLs for the leaf index (LI), and these QTLs were located in LG10 from 45.967 cM to 73.823 cM (27.856 cM), including 44 SNPs. The largest LOD (13.13) and PVE (50.5%) values were observed for *qLI10-4* and *qLI10-12*, respectively (Fig. 6). Some LI QTLs in LG10 were gathered tightly with an average interval of less than 0.63 cM per marker, indicating that these QTLs might belong to the same QTL (Fig. 6). For LL, two QTLs (*qLL9-1* and *qLL9-2*) were mapped to LG09 and supported by 16 SNPs. One stable FW QTL was anchored to LG10 with a PVE value of 59.2% but supported by one marker. Two stable FL QTLs were anchored to LG10 and LG12 (*qFL10* and *qFL12*) with nine significantly linked SNPs, explaining 7.3% to 36.9% of the phenotypic variation (Supplementary Table 3). Five QTLs (*qFI10-1* ~ *qFI10-5*) at 37.344 ~ 66.309 cM in LG10 accounted for 15.8% to 51.9% of the PVE, with the highest LOD value (more than 5.0) in these QTL regions. Notably, *qFI10-4* was detected repeatedly in 2017 and 2018 (Fig. 6). Two stable QTLs (*qFI*7-1 and *qFI7-2*) for the FI at 60.634 ~ 61.916 cM in LG07 accounted for 8.5% and 19.3% of the PVE, respectively, with an average intermarker distance of 0.18 cM. We extracted predicted genes within 150 kb upstream and downstream of the markers in stable QTLs. To further explore the expression of stable QTLs for fruit-related traits, we performed RNA-seq and found that 82 out of 188 predicted genes showed differential expression (Supplementary Table 4). These genes represent valuable resources for further gene cloning and marker-assisted selection (MAS).

## Discussion

A high-density genetic map can provide valuable information for deciphering the genetic basis of important and complex agronomic traits. With the rapid development of sequencing technology, SNPs and InDels have been mined for high-density linkage map construction, QTL dissection, candidate gene discovery and breeding applications in crops [10, 15, 16]. Whole-genome resequencing for SNP genotyping in biparental segregation populations has been successfully applied to plants [17–19]. Because of our complete de novo assembled genome sequence of *Lycium* (Cao et al., unpublished 2021), it has become possible to use whole-genome resequencing for genome-wide SNP discovery and high-density genetic map construction in *Lycium*. In this study, whole-genome resequencing resulted in 9,015,078 SNPs and 1,646,131 InDels, and a high-density genetic map containing 8,507 SNPs spanning 2,122.24 cM with an average distance of 0.25 cM between adjacent markers was constructed based on a hybrid F_1_ population (NY map). However, the maximum gap in the integrated map was 19.41 cM, in LG 11. This indicated that there was recombination or undeveloped markers in this area [20]. Compared with that in the first sequencing-based genetic map of *Lycium* that we constructed [14], the number of F_1_ individuals was reduced by 100 (the number in the first population containing 302 individuals), whereas the average distance was very similar to that of the published genetic map. Nevertheless, the total number of SNPs and total map distance of this resequencing-based linkage map were higher, indicating higher resolution for resequencing than for reduced-representation sequencing. The consensus map developed by integrating these two high-density maps indicated that a saturated genetic map had been obtained for *Lycium*.

High-density genetic maps can improve contig/scaffold assembly at the chromosome level [21–23]. Generally, a genetic map can distribute 60–90% of all the assembled contigs/scaffolds on pseudochromosomes [24–26]. Given the increased marker density, the distribution rate of the consensus genetic map will increase accordingly [23, 27]. In this study, a high-density genetic map of *Lycium* was constructed on the basis of resequencing, and a consensus map was produced by integrating the high-density genetic map and a previously published map [14]. The final genetic map contained 15,240 markers in 12 LGs. Based on this consensus genetic map, the scaffold *Lycium* genome (Cao et al., unpublished 2021) was mounted onto 12 pseudochromosomes with a distribution rate of 64.30%, which is relatively low. There were 783,254 unmounted scaffolds, which was 476.72 times the number of anchored scaffolds. In addition, the average length of the unplaced scaffolds was 0.86 kb. Therefore, we speculate that a fragmented genome assembly is the cause of the low distribution rate. For the *Lycium* genome used in this study, the distribution rate could benefit from the use of PacBio long-read sequencing [28], a BioNano optical map [24], or Hi-C technology [29].

To gain insights into the evolutionary history of wolfberry, we conducted collinearity analyses on the genomes of wolfberry, tomato, pepper, and potato. Similar to previous results [14], the genetic map of wolfberry showed low collinearity with these three genomes overall, suggesting greater differentiation of wolfberry. However, high collinearity was observed in some cases, such as segments on chromosomes 02, 04, 06, 10, and 11 of wolfberry for wolfberry-pepper pairs and wolfberry-tomato pairs, suggesting that these intervals are highly conserved among different species of Solanaceae. In addition, marker density was associated with collinearity as more markers meant a greater probability of markers aligning to the corresponding reference genome sequences. The markers on a high-density genetic map were typically developed by reduced-representation sequencing [14, 30] or re-sequencing [17–19, 31], which could yield thousands of randomly distributed markers. But the reference genomes of plants always harbor hundreds of Mbp at least. So only the collinearity of conserved regions could be detected using a sequencing-based high density genetic map and we believe that a comprehensive genomic synteny could be realized using a genome assembly of wolfberry.

Fruits and leaves are the main medicinal and edible parts of *Lycium* spp. [32]. In our study, many QTLs (Supplementary Table 2) were identified for fruit and leaf traits based on phenotypic data collected over 3 years. Most of the measured traits were located at more than two QTLs, with QTLs for the FI mapped to LG04, LG09, LG11, and LG12 at different positions. In addition, we found a large number of QTLs linked to two or three traits, with those for the FW, FL and FI traits mapped to 37.344 cM in LG10 with LOD and PVE values up to 16.02 and 62.5%, respectively. These results indicated that fruit and leaf traits in *Lycium* are controlled by multiple loci and single loci with pleiotropic effects [33].

Stable QTLs are valuable and useful for MAS-based breeding programs and have been identified in perennials [34]. In this study, 25 stable QTLs associated with two leaf and three fruit traits were identified in LG07, LG09, LG10, and LG11 (Supplementary Table 3), four of which were identified in two LG10 regions (38.6 cM and 66.3 cM) with a high LOD value (15.7– 45.3) and were related to three traits (LI, FW, and FI). In addition, in a previous study [14], two stable QTLs for FW were located in the same two LG regions (133.6 cM and 146.4 cM) as two stable QTLs for FD, which might indicate that these stable QTLs (*qLI10-10*, *qFW10*, *qFI10-2,* and *qFI10-5*) show high reliability in different environments and should be considered major QTLs. Of note, given these major QTLs, there is a genetic basis for the phenotypic correlation between FW and the FI, which is consistent with the strong correlation observed between the two traits in phenotypic analyses (Fig. 1A-C). Therefore, we speculate that two regions (38.6 cM and 66.3 cM) in LG10 play crucial roles in regulating *Lycium* fruit growth and development. The SNPs underlying these QTLs could be verified using the 100 remaining F_1_ individuals in the future, and the tightly linked SNPs could be converted into kompetitive allele-specific PCR (KASP) markers and potentially used as early selection markers.

## Methods

### Mapping population construction and phenotyping

A hybrid wolfberry population derived from a cross between ‘Ningqi No. 1’ (NN) (*L. barbarum* L.) and ‘Yunnan Gouqi’ (YG) (*Lycium yunnanense* Kuang et A.M. Lu) was generated in August 2014. The female parent NN is a major artificial breeding cultivar in Northwest China. Its fruit is bright red and elliptical with a sweet taste, and its leaves are lanceolate. The male parent YG is a wild-type wolfberry with dark red, long, oval, bitter-tasting fruit (Fig. 1). Seeds of the F_1_ hybrid and the two parents were collected and sown in the Ningxia Academy of Agriculture and Forestry Sciences National Wolfberry Germplasm Resources Garden (38°38 N, 106°90 E), Yinchuan City, Hui Autonomous Region, Ningxia, China, in May 2015. In total, 300 F_1_ individuals were grown, 200 of which were randomly selected to establish the mapping population. Water and fertilizer management was the same as that used in the production field. Weeds were managed manually.

The leaf- and fruit-related traits were measured in the F_1_ population (NY) and the two parents. LL was the maximum distance between the leaf base and tip. LW was the widest distance across the leaf. FW was the weight of one mature fruit. FL was the maximum distance between the top and bottom of the fruit. FD was the widest distance across a fruit. LL, LW, FW, FL, and FD were measured according to methods described elsewhere [35]. LL, LW, FL, and FD were measured using Vernier calipers, whereas FW was acquired using an electronic balance (SE602F, Ohaus, NJ, USA). LI and FI were calculated according to Equations (i) and (ii): (i) LI = LL/LW [35] and (ii) FI = FL/FD [35]. In total, 30 leaves and fruits collected from each tree for 3 consecutive years (from 2016 to 2018) were used to obtain phenotypic data. The average values of each trait per individual were used for QTL analysis. Complex variance analysis, variance analysis, and correlation analysis were carried out using SPSS V17.0 software (SPSS Inc., Chicago, IL, USA).

### Population resequencing and genotyping

Genomic DNA was extracted from the young leaves of both parents and 200 F_1_ plants using a Super Plant Genomic DNA DP360 Kit (Tiangen Biotech, Beijing, China). DNA concentration was measured using a NanoDrop spectrophotometer (ND2000, Thermo Fisher Scientific, USA), and DNA quality was monitored by electrophoresis on 0.85% agarose gels. The genomic DNA was sheared into 350-bp fragments using an S2/E210 Ultrasonicator (Covaris, USA). The purified products were then ligated for end repair, subjected to 3’A and adaptor addition, and selected according to fragment size on a 1% agarose gel. The final library was constructed by PCR. Library quantification was performed using an Agilent 2100 Bioanalyzer (Agilent Technologies, Palo Alto, CA, USA), and the fragments of the libraries were paired-end sequenced (PE125) using the Illumina XTen platform (Illumina, San Diego, CA, USA) according to the manufacturer’s recommendations.The data that support the findings of this study have been deposited into CNGB Sequence Archive (CNSA) of China National GeneBank DataBase (CNGBdb) with accession number CNP0001536.

Raw reads were filtered to generate high-quality clean reads by (i) removing adaptor sequences, (ii) filtering reads with > 10% unidentified nucleotides, and (iii) removing reads with > 50% bases with a low Phred quality score (≤ 10). Burrows-Wheeler Aligner [36] was used to align the clean reads to the *Lycium* genome (Cao et al., unpublished 2021), and duplicates were identified using Picard (Picard: http://sourceforge.net/projects/picard/). SNPs and insertions and deletions (InDels; 1–5 bp) were called using GATK software [37] and then annotated by SnpEff [38]. Genome variation maps of SNPs and InDels were drawn by Circos [39]. SNP genotypes were encoded using biallelic coding rules and eight genotyping patterns (aa × bb, ab × cd, ef × eg, hk × hk, lm × ll, nn × np, ab × cc, and cc × ab). All patterns except aa × bb were selected to construct a high-density genetic map for a cross-pollinated (CP) population.

### Genetic linkage map construction and QTL mapping

The resulting SNPs were further screened. Specifically, hk × hk and nn × np segregation-type SNPs with a depth < 6 × in the parents, an integrity < 60%, and significant segregation distortion (SD) (Chi-square *P* < 0.05) were filtered out, whereas the remaining segregation types with a depth < 10 × in the parents, an integrity < 70%, and highly significant SD (chi-square *P* < 0.01) were filtered out. SNP markers were arranged into linkage groups (LGs) based on pairwise modified logarithm of odds (MLOD) scores. Markers with MLOD scores > 5 were assigned to a single LG. SMOOTH algorithms [40] were used to correct genotypes, and then the k-nearest neighbor approach [41] was used for genotype imputation. The JoinMap software (V4.1) mapping function for the CP population type was applied for linear arrangement within LGs. Map distances were estimated using the Kosambi mapping function [42]. A heat map of adjacent SNP relationships was generated using R (www.r-project.org/). MapQTL V6.0 with the interval mapping (IM) model was used for QTL analyses [43], and the LOD threshold value was set to 2.5. QTLs with a threshold LOD value > 3.0 and PVE > 10% were considered major QTLs.

### Genetic map-assisted genome scaffold assembly and genome synteny analyses

SLAF markers of the published genetic map (NC map) [14] and SNPs identified in this study were aligned to the *Lycium* scaffold-level genome assembly (Cao et al., unpublished 2021) using BLAT software [44]. The corresponding relationship and shared markers of these two LGs were extracted according to the locations of SLAF and SNP markers. An integrated genetic map was constructed using BioMercator v4.2 [45]. This integrated genetic map was used to anchor the scaffolds of the *Lycium* genome at the chromosome level using ALLMAPS software [46]. The SNPs integrated genetic map were aligned to the genomes of solanaceous relatives, namely, pepper (http://peppersequence.genomics.cn/page/species/download.jsp #5), tomato (ftp://ftp.solgenomics.net/tomato_genome/assembly/build_2.50/), and potato (http://solanaceae.plantbiology. msu.edu /pgsc_download.shtml), using BLAT [44] and the physical positions of the homologous sequence were used to generate a collinearity diagram in R.

### RNA-seq analysis

The fruits of the two parents were sampled in triplicate 36–40 days after pollination for total RNA extraction [RNAprep Pure Plant Kit (Tiangen Biotech, Beijing, China)]. An RNA-seq library was constructed according to [47]; the library was then loaded into Cbot for cluster generation and for 150-bp paired-end read sequencing on the Illumina NovaSeq platform (Illumina, San Diego, CA, USA). Low-quality reads [> 20% of bases with a Q value ≤ 20 or an ambiguous sequence content (‘N’) exceeding 5%] were removed by an in-house Perl script. The clean reads were then mapped to the reference genome of wolfberry (Cao et al., unpublished 2021) using STAR with default settings [48]. StringTie was used to assemble transcripts [49]. The fragments per kilobase of transcript per million mapped reads (FPKM) method was used to quantify transcript expression levels, and the DESeq R package was used to detect differentially expressed genes (DEGs). Significant DEGs were identified using an adjusted *P* value < 0.05 and a fold change ≥ 2.

## Supplementary Information

**Additional file 1: Figure 1**. Two genetic map. (A) The high-density genetic map by resequencing; (B) The consensus genetic map.

**Additional file 2: Figure 2. **Genetic map-assisted genome assembly from LG01 to LG12 except LG08.

**Additional file 3: Table 1. **Data generated during resequencing.

**Additional file 4: Table 2.** QTLs detected in the F_1_ population.

**Additional file 5: Table 3.** Stable QTLs.

**Additional file 6: Table 4.** Annotation of differentially expressed genes underlying QTLs for fruit-related traits.

## Data Availability

All the sequencing clean data were uploaded to the China National GeneBank DataBase (CNP0001536). However, the data will be made public on 1 April 2022. Before 1 April 2022, the datasets are available from the corresponding author on reasonable request.

## Abbreviations

SNPs: Single-nucleotide polymorphisms
QTLs: Quantitative trait loci
LOD: Logarithm of odds
NN: ‘Ningqi No. 1’
YG: ‘Yunnan Gouqi’
NC: ‘Ningqi No. 1’ × ‘Chinese Gouqi’
NY: ‘Ningqi No. 1’ × ‘Yunnan Gouqi’
MAS: Marker-assisted selection
NGS: Next-generation sequencing
LL: Leaf length
LW: Leaf width
LI: Leaf index
FW: Single-fruit weight
FL: Fruit longitude
FD: Fruit diameter
FI: Fruit index
CDS: Coding sequence
LGs: Linkage groups
SD: Segregation distortion
PVE: Phenotypic variance explained
KASP: Kompetitive allele-specific PCR
CNGBdb: China National GeneBank DataBase
CP: Cross-pollinated
MLOD: Modified logarithm of odds
FPKM: Fragments per kilobase of transcript per million mapped reads
DEGs: Differentially expressed genes
