# Supplementary material for: A consensus and saturated genetic map provides insight into genome anchoring, synteny of Solanaceae and leaf- and fruit-related QTLs in wolfberry (Lycium Linn.)
Source: BMC Plant Biol. 2021 Jul 24;21:350. doi: 10.1186/s12870-021-03115-1 (PMC8306383; doi:10.1186/s12870-021-03115-1)
Supplement: Supplementary file 2 — Additional file 2: Figure 2. Genetic map-assisted genome assembly from LG01 to LG12 except LG08. [file 12870_2021_3115_MOESM2_ESM.pdf]

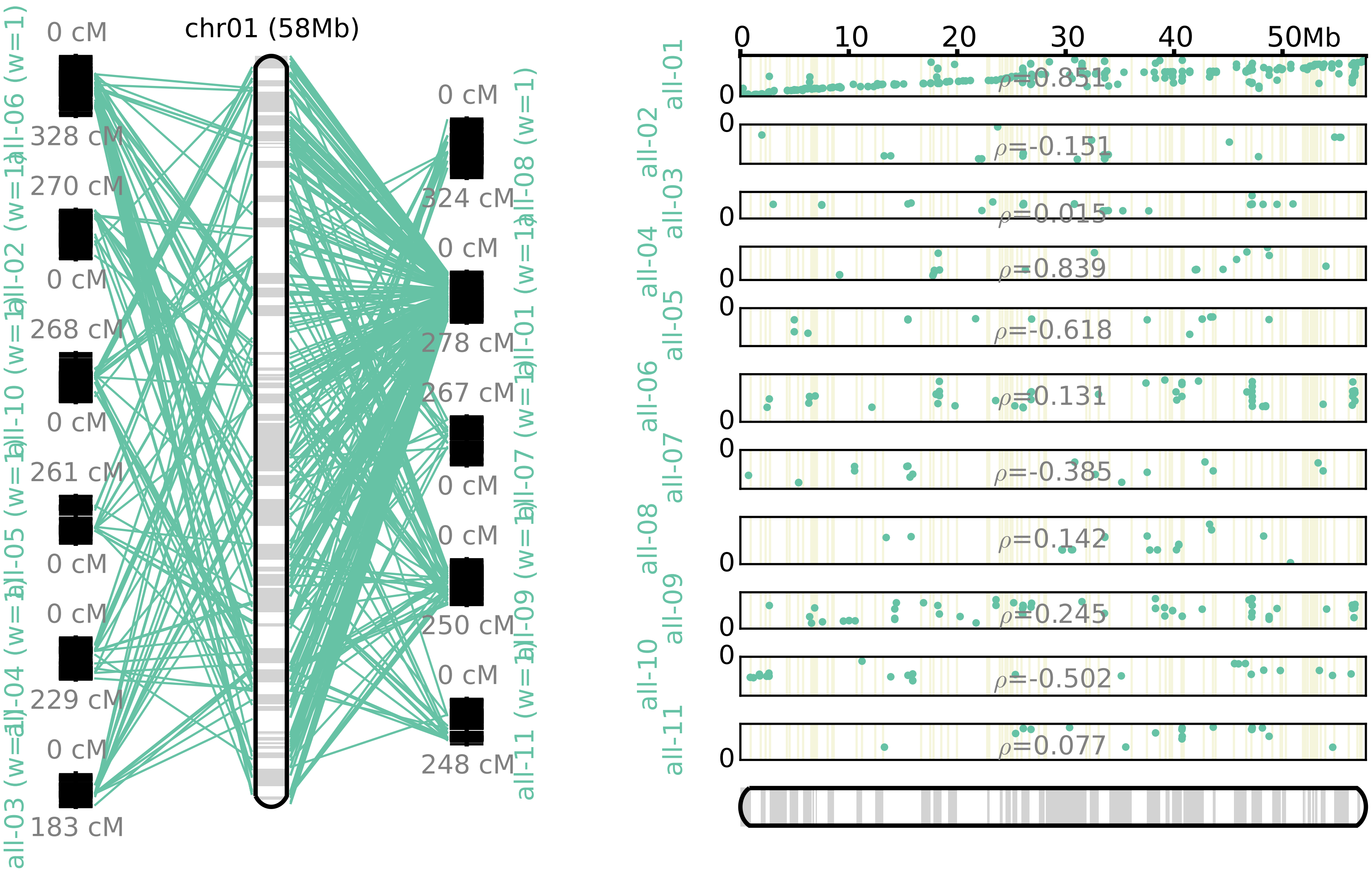
chr01 (58Mb)
all-06 (w=1)
0 cM
328 cM
all-02 (w=1)
270 cM
0 cM
all-10 (w=1)
268 cM
0 cM
all-05 (w=1)
261 cM
0 cM
all-04 (w=1)
0 cM
229 cM
all-03 (w=1)
0 cM
183 cM
all-08 (w=1)
0 cM
324 cM
all-01 (w=1)
0 cM
278 cM
all-07 (w=1)
267 cM
0 cM
all-09 (w=1)
0 cM
250 cM
all-11 (w=1)
0 cM
248 cM
0
10
20
30
40
50Mb
all-01
ρ=0.851
all-02
ρ=-0.151
all-03
ρ=0.015
all-04
ρ=0.839
all-05
ρ=-0.618
all-06
ρ=0.131
all-07
ρ=-0.385
all-08
ρ=0.142
all-09
ρ=0.245
all-10
ρ=-0.502
all-11
ρ=0.077

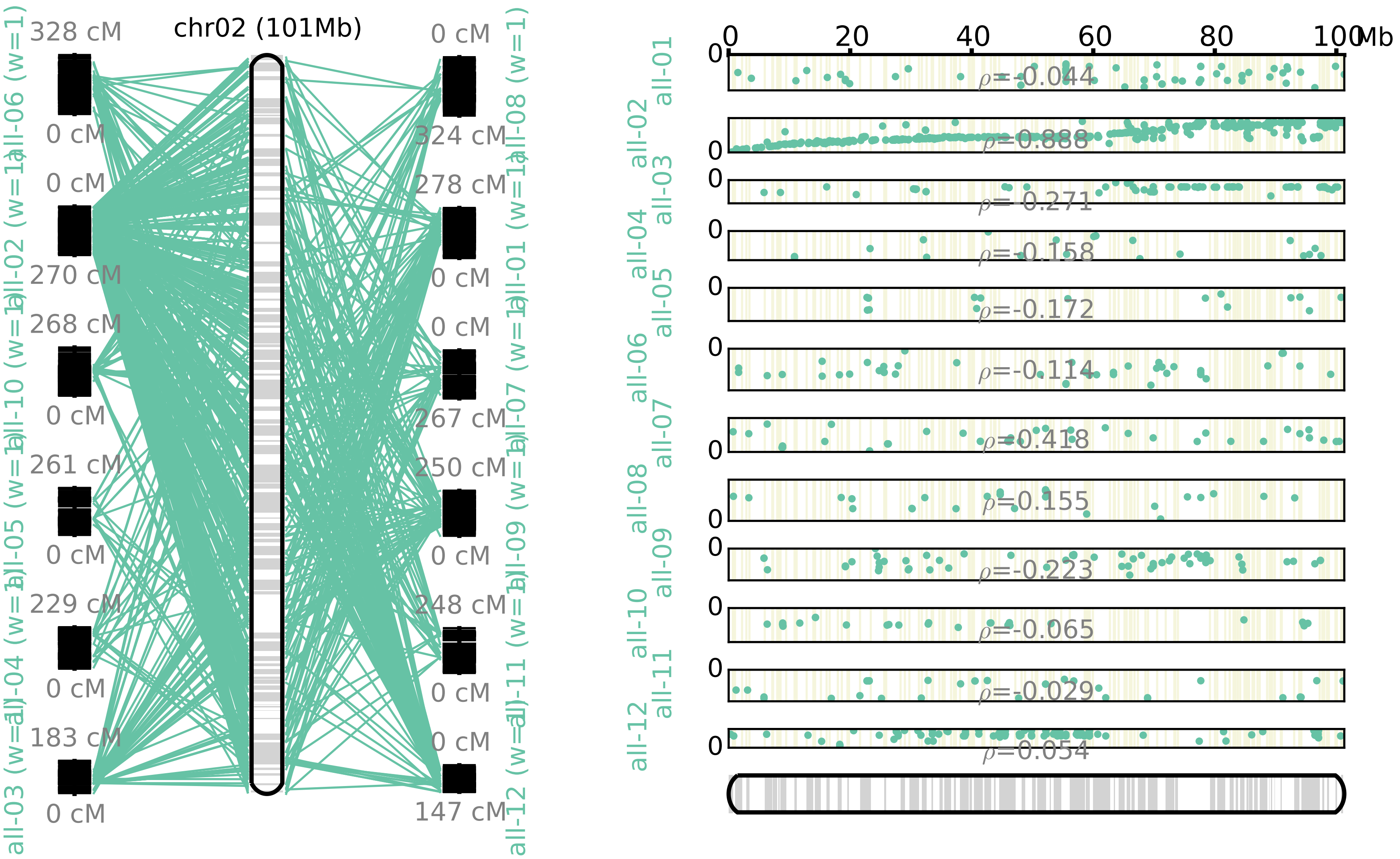
chr02 (101Mb)
all-06 (w=1)
328 cM
0 cM
all-02 (w=1)
0 cM
270 cM
all-10 (w=1)
268 cM
0 cM
all-05 (w=1)
261 cM
0 cM
all-04 (w=1)
229 cM
0 cM
all-03 (w=1)
183 cM
0 cM
all-08 (w=1)
0 cM
324 cM
all-01 (w=1)
278 cM
0 cM
all-07 (w=1)
0 cM
267 cM
all-09 (w=1)
250 cM
0 cM
all-11 (w=1)
248 cM
0 cM
all-12 (w=1)
0 cM
147 cM
0
20
40
60
80
100
Mb
all-01
ρ=-0.044
all-02
ρ=0.888
all-03
ρ=-0.271
all-04
ρ=-0.158
all-05
ρ=-0.172
all-06
ρ=-0.114
all-07
ρ=0.418
all-08
ρ=0.155
all-09
ρ=-0.223
all-10
ρ=-0.065
all-11
ρ=-0.029
all-12
ρ=0.054

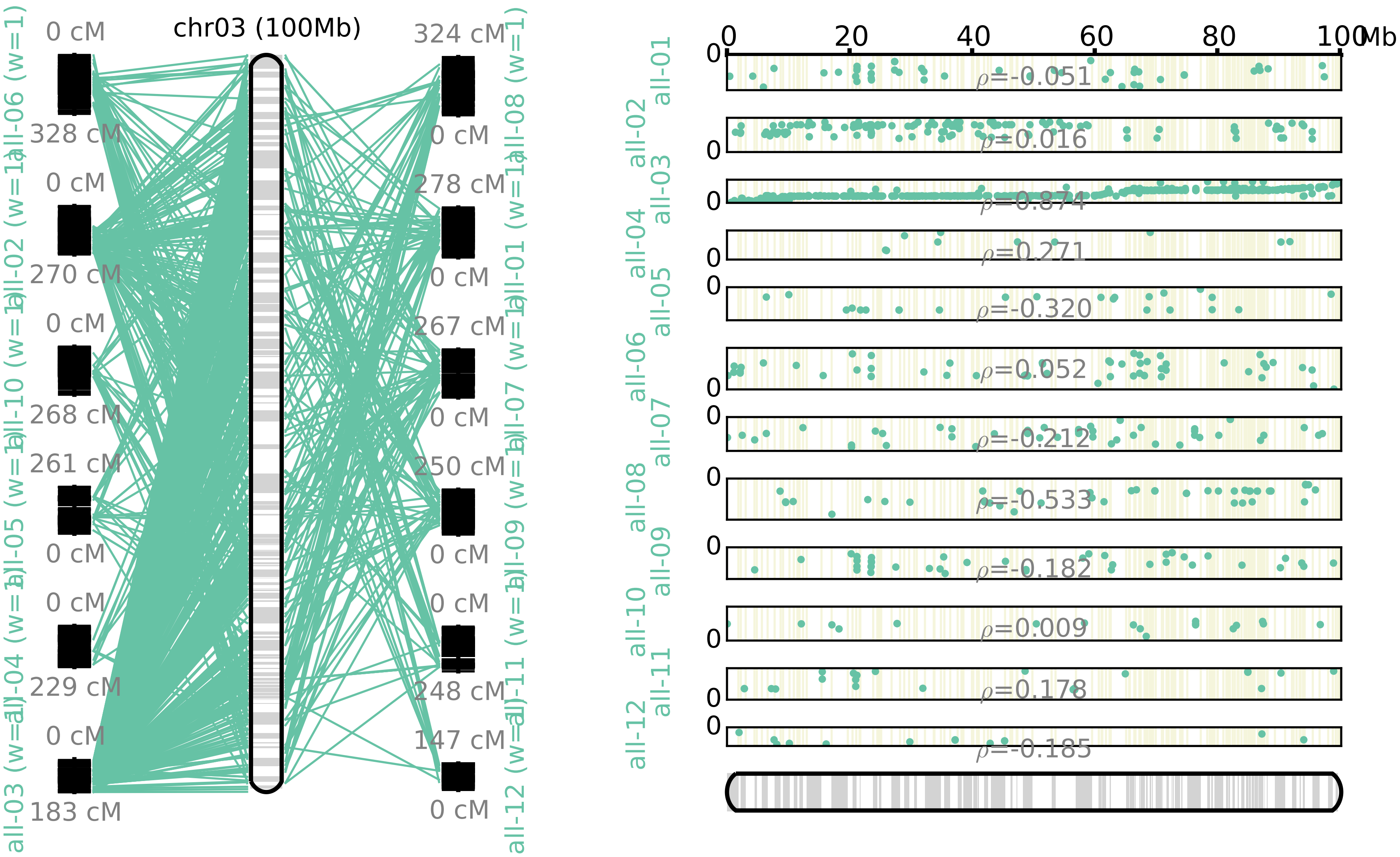
chr03 (100Mb)
all-06 (w=1)
all-02 (w=1)
all-10 (w=1)
all-05 (w=1)
all-04 (w=1)
all-03 (w=1)
all-08 (w=1)
all-01 (w=1)
all-07 (w=1)
all-09 (w=1)
all-11 (w=1)
all-12 (w=1)
0 cM
328 cM
270 cM
268 cM
261 cM
229 cM
183 cM
324 cM
278 cM
267 cM
250 cM
248 cM
147 cM
0
20
40
60
80
100
Mb
all-01
all-02
all-03
all-04
all-05
all-06
all-07
all-08
all-09
all-10
all-11
all-12
ρ=-0.051
ρ=0.016
ρ=0.874
ρ=0.271
ρ=-0.320
ρ=0.052
ρ=-0.212
ρ=-0.533
ρ=-0.182
ρ=0.009
ρ=0.178
ρ=-0.185

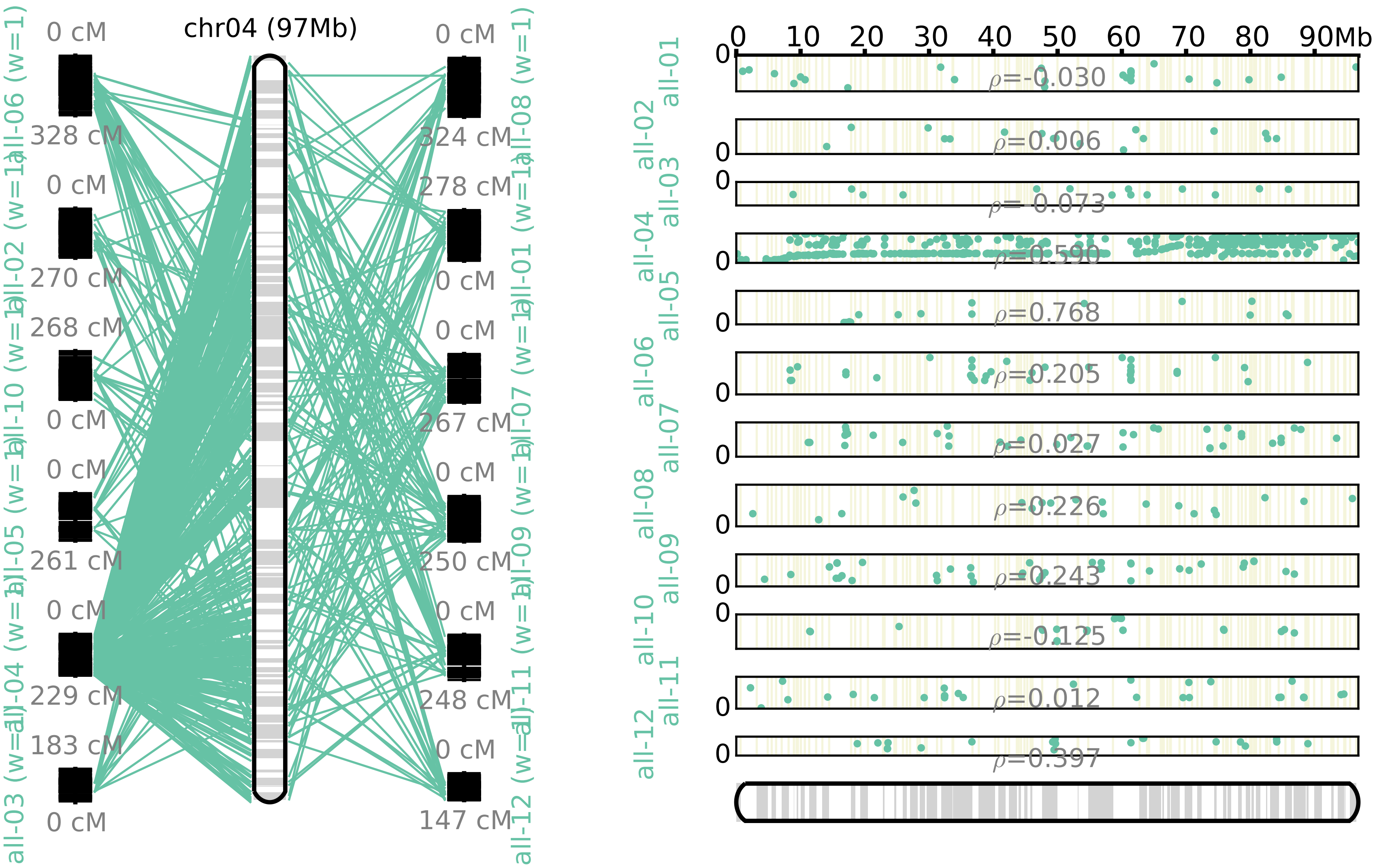

chr04 (97Mb)
0 cM
328 cM
270 cM
268 cM
261 cM
229 cM
183 cM
324 cM
278 cM
267 cM
250 cM
248 cM
147 cM
all-06 (w=1)
all-02 (w=1)
all-10 (w=1)
all-05 (w=1)
all-04 (w=1)
all-03 (w=1)
all-08 (w=1)
all-01 (w=1)
all-07 (w=1)
all-09 (w=1)
all-11 (w=1)
all-12 (w=1)
0 10 20 30 40 50 60 70 80 90Mb
all-01
all-02
all-03
all-04
all-05
all-06
all-07
all-08
all-09
all-10
all-11
all-12
ρ=-0.030
ρ=0.006
ρ=-0.073
ρ=0.590
ρ=0.768
ρ=0.205
ρ=0.027
ρ=0.226
ρ=0.243
ρ=-0.125
ρ=0.012
ρ=0.397

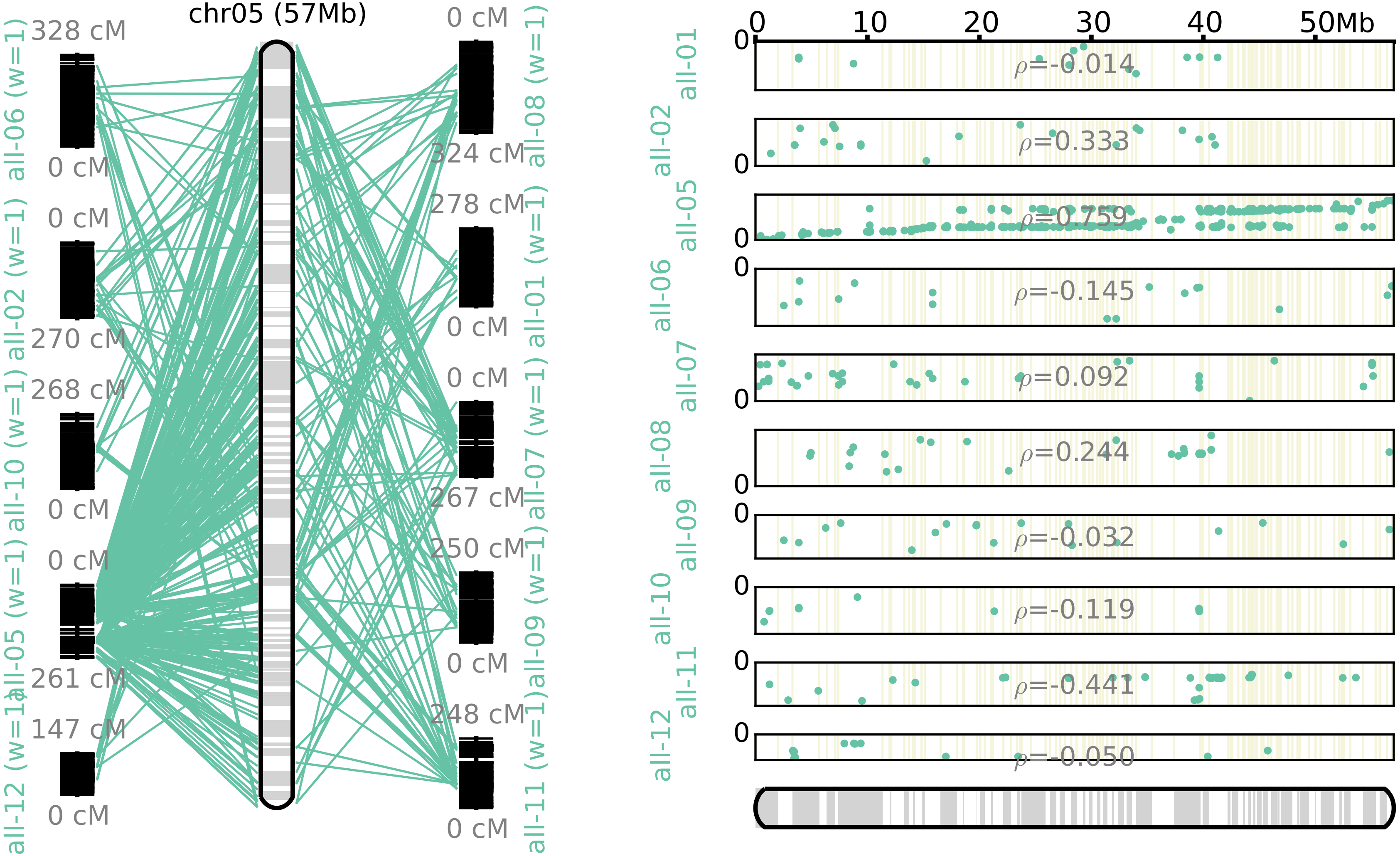
chr05 (57Mb)
all-06 (w=1)
328 cM
0 cM
all-02 (w=1)
0 cM
270 cM
all-10 (w=1)
268 cM
0 cM
all-05 (w=1)
0 cM
261 cM
all-12 (w=1)
147 cM
0 cM
all-08 (w=1)
0 cM
324 cM
all-01 (w=1)
278 cM
0 cM
all-07 (w=1)
0 cM
267 cM
all-09 (w=1)
250 cM
0 cM
all-11 (w=1)
248 cM
0 cM
0
10
20
30
40
50Mb
all-01
ρ=-0.014
all-02
ρ=0.333
all-05
ρ=0.759
all-06
ρ=-0.145
all-07
ρ=0.092
all-08
ρ=0.244
all-09
ρ=-0.032
all-10
ρ=-0.119
all-11
ρ=-0.441
all-12
ρ=-0.050

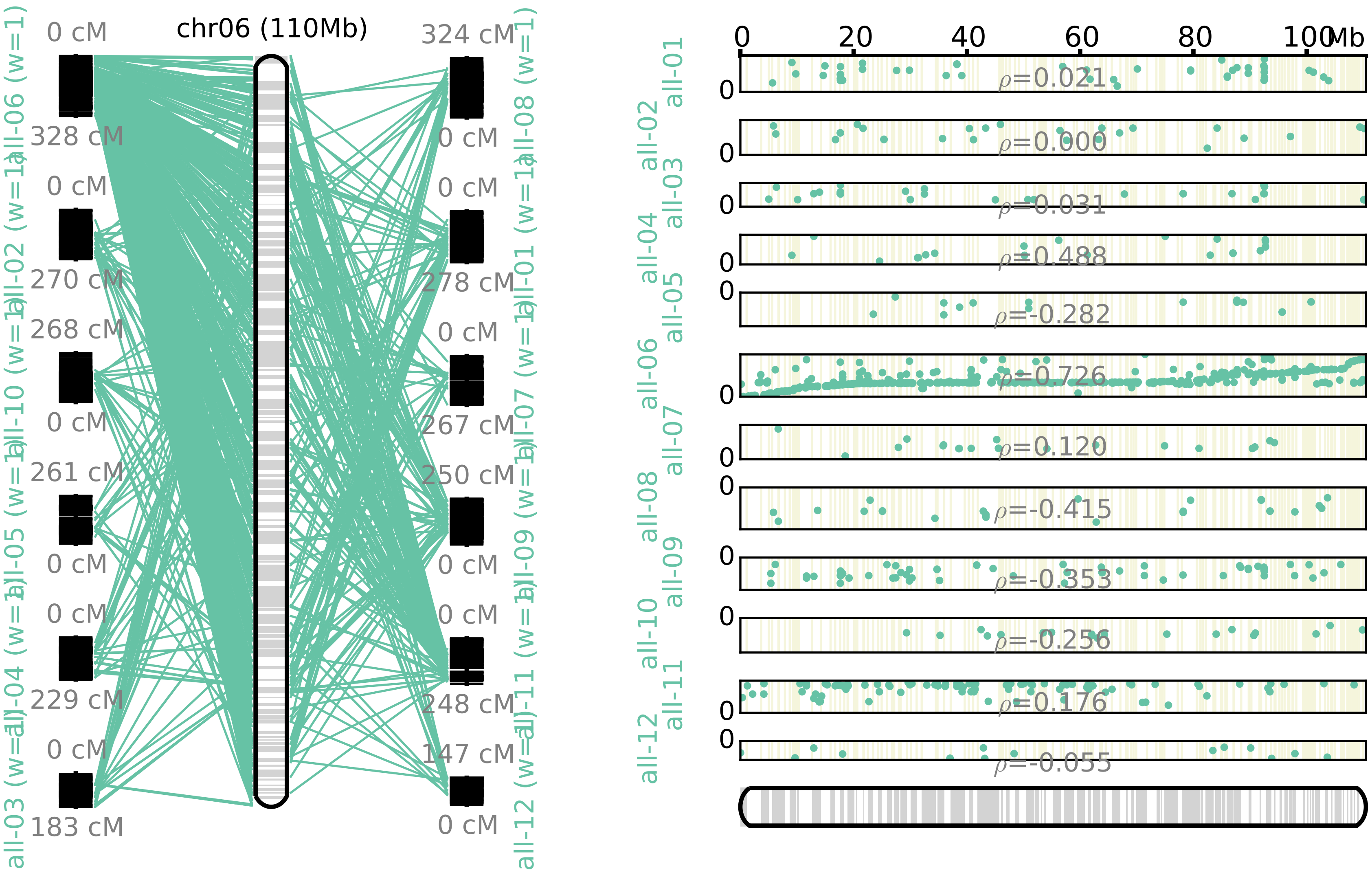
chr06 (110Mb)
all-06 (w=1)
all-02 (w=1)
all-10 (w=1)
all-05 (w=1)
all-04 (w=1)
all-03 (w=1)
all-08 (w=1)
all-01 (w=1)
all-07 (w=1)
all-09 (w=1)
all-11 (w=1)
all-12 (w=1)
0 cM
328 cM
270 cM
268 cM
261 cM
229 cM
183 cM
324 cM
278 cM
267 cM
250 cM
248 cM
147 cM
0
20
40
60
80
100
Mb
all-01
all-02
all-03
all-04
all-05
all-06
all-07
all-08
all-09
all-10
all-11
all-12
ρ=0.021
ρ=0.000
ρ=0.031
ρ=0.488
ρ=-0.282
ρ=0.726
ρ=0.120
ρ=-0.415
ρ=-0.353
ρ=-0.256
ρ=0.176
ρ=-0.055

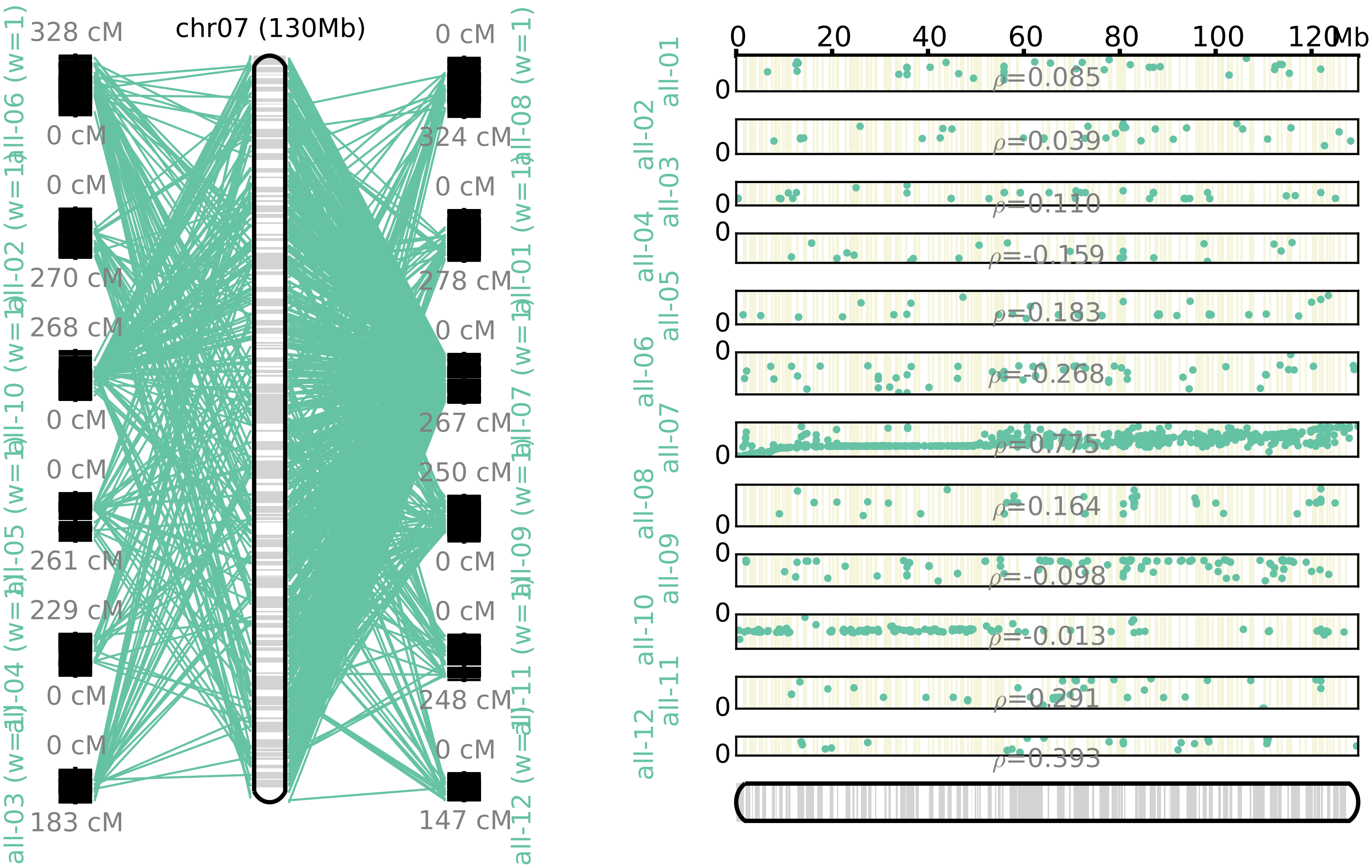

chr07 (130Mb)
all-06 (w=1)
328 cM
0 cM
all-02 (w=1)
0 cM
270 cM
all-10 (w=1)
268 cM
0 cM
all-05 (w=1)
0 cM
261 cM
all-04 (w=1)
229 cM
0 cM
all-03 (w=1)
0 cM
183 cM
all-08 (w=1)
0 cM
324 cM
all-01 (w=1)
0 cM
278 cM
all-07 (w=1)
0 cM
267 cM
all-09 (w=1)
250 cM
0 cM
all-11 (w=1)
0 cM
248 cM
all-12 (w=1)
0 cM
147 cM
0
20
40
60
80
100
120
Mb
all-01
ρ=0.085
all-02
ρ=0.039
all-03
ρ=0.110
all-04
ρ=-0.159
all-05
ρ=0.183
all-06
ρ=-0.268
all-07
ρ=0.775
all-08
ρ=0.164
all-09
ρ=-0.098
all-10
ρ=-0.013
all-11
ρ=0.291
all-12
ρ=0.393

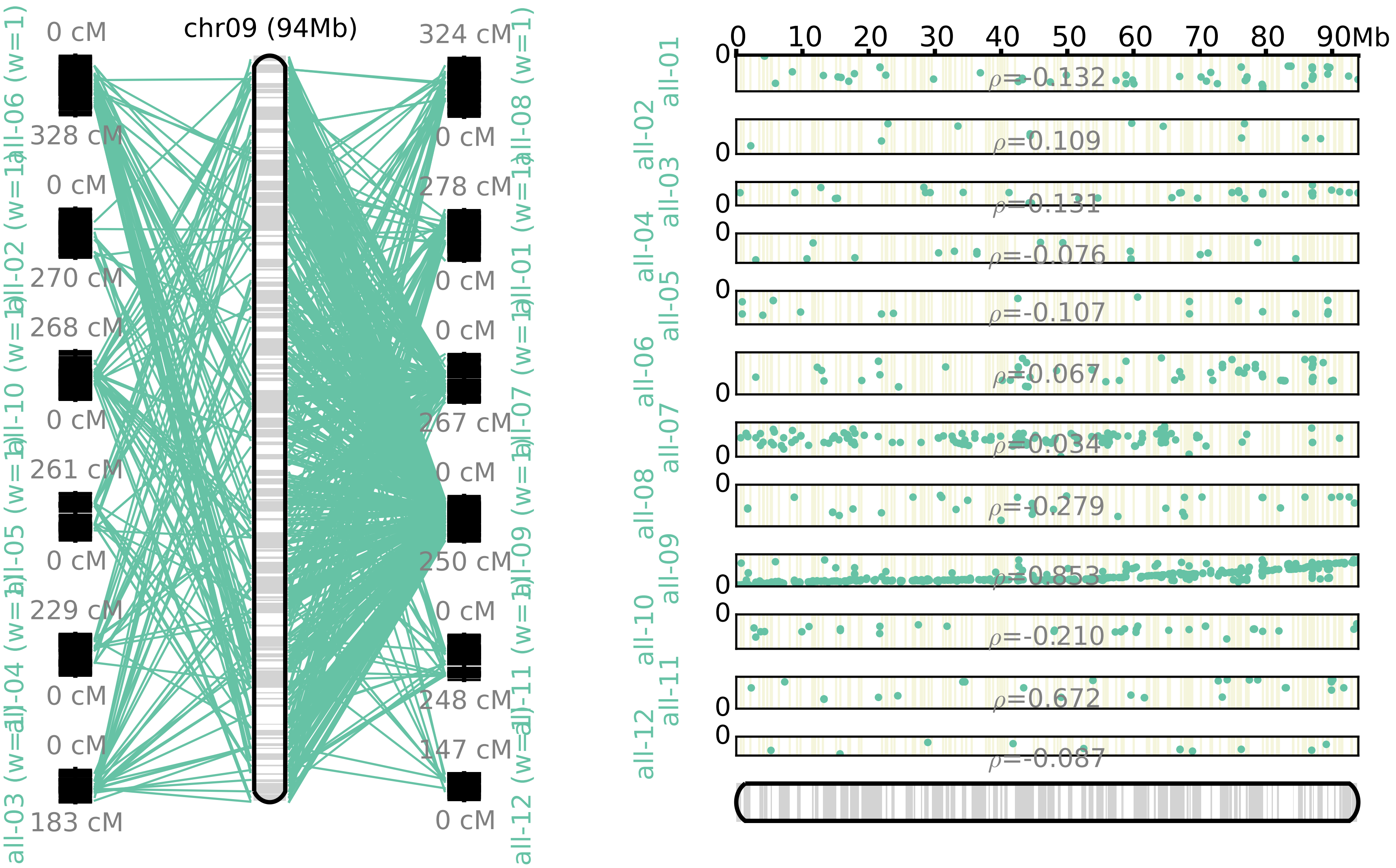

chr09 (94Mb)
0 cM
328 cM
270 cM
268 cM
261 cM
229 cM
183 cM
324 cM
278 cM
267 cM
250 cM
248 cM
147 cM
all-06 (w=1)
all-02 (w=1)
all-10 (w=1)
all-05 (w=1)
all-04 (w=1)
all-03 (w=1)
all-08 (w=1)
all-01 (w=1)
all-07 (w=1)
all-09 (w=1)
all-11 (w=1)
all-12 (w=1)
0 10 20 30 40 50 60 70 80 90Mb
all-01
all-02
all-03
all-04
all-05
all-06
all-07
all-08
all-09
all-10
all-11
all-12
ρ=-0.132
ρ=0.109
ρ=0.131
ρ=-0.076
ρ=-0.107
ρ=0.067
ρ=0.034
ρ=-0.279
ρ=0.853
ρ=-0.210
ρ=0.672
ρ=-0.087

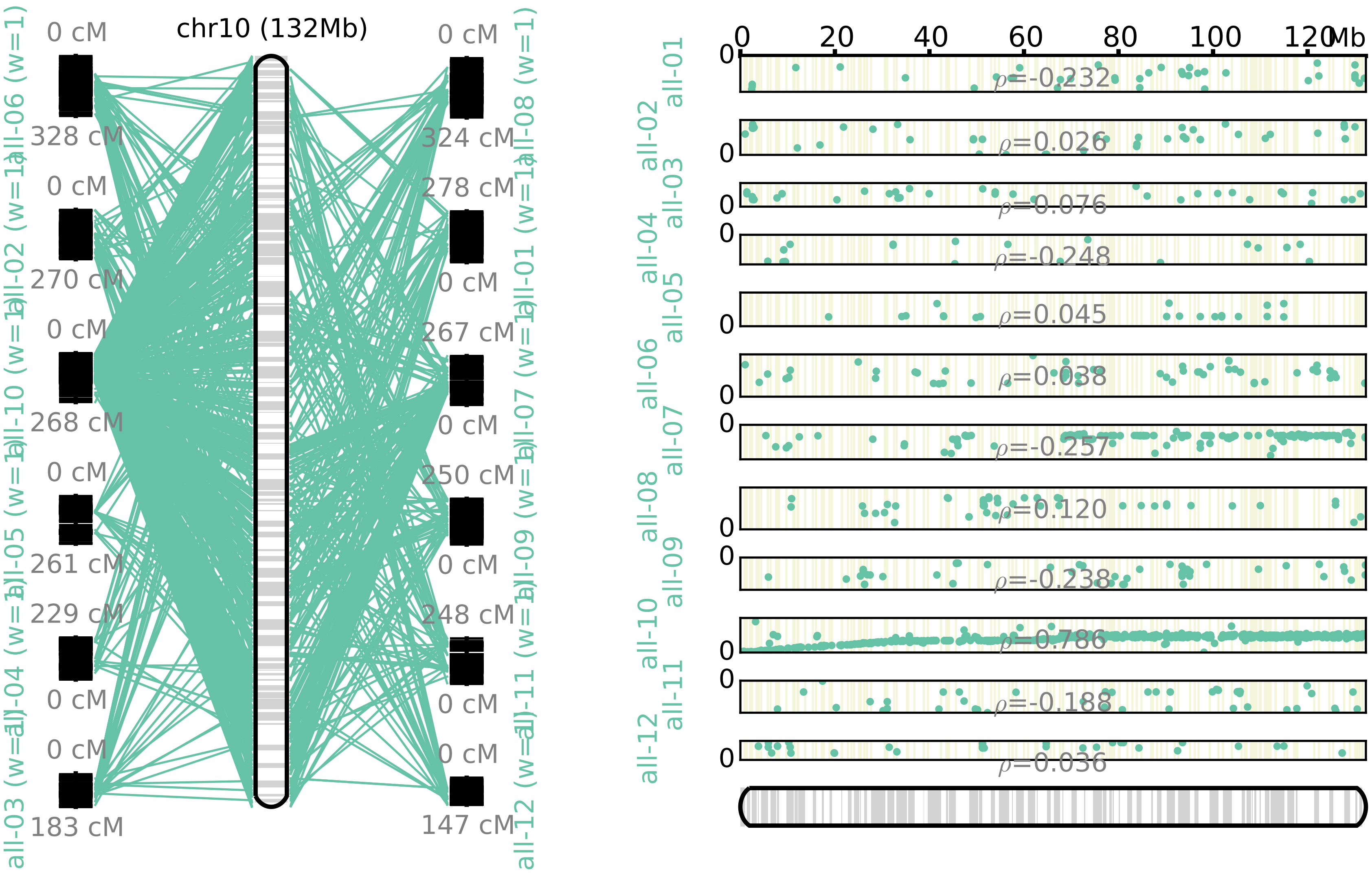

chr10 (132Mb)
all-06 (w=1)
0 cM
328 cM
all-02 (w=1)
0 cM
270 cM
all-10 (w=1)
0 cM
268 cM
all-05 (w=1)
0 cM
261 cM
all-04 (w=1)
229 cM
0 cM
all-03 (w=1)
0 cM
183 cM
all-08 (w=1)
0 cM
324 cM
all-01 (w=1)
278 cM
0 cM
all-07 (w=1)
267 cM
0 cM
all-09 (w=1)
250 cM
0 cM
all-11 (w=1)
248 cM
0 cM
all-12 (w=1)
0 cM
147 cM
0
20
40
60
80
100
120
Mb
all-01
ρ=-0.232
all-02
ρ=0.026
all-03
ρ=0.076
all-04
ρ=-0.248
all-05
ρ=0.045
all-06
ρ=0.038
all-07
ρ=-0.257
all-08
ρ=0.120
all-09
ρ=-0.238
all-10
ρ=0.786
all-11
ρ=-0.188
all-12
ρ=0.036

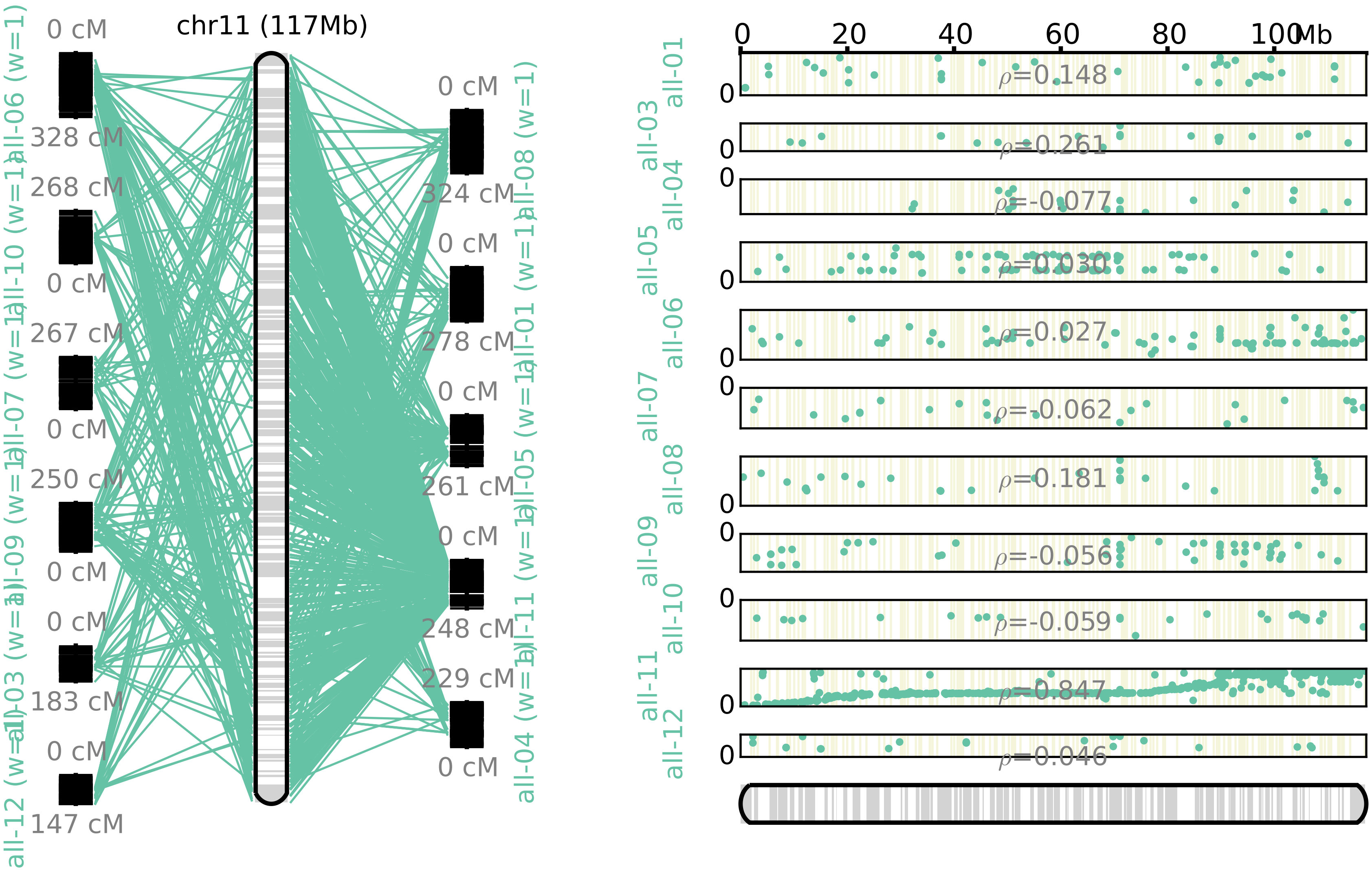
chr11 (117Mb)
all-06 (w=1)
0 cM
328 cM
all-10 (w=1)
268 cM
0 cM
all-07 (w=1)
267 cM
0 cM
all-09 (w=1)
250 cM
0 cM
all-03 (w=1)
0 cM
183 cM
all-12 (w=1)
0 cM
147 cM
all-08 (w=1)
0 cM
324 cM
all-01 (w=1)
0 cM
278 cM
all-05 (w=1)
0 cM
261 cM
all-11 (w=1)
0 cM
248 cM
all-04 (w=1)
229 cM
0 cM
0
20
40
60
80
100
Mb
all-01
ρ=0.148
all-03
ρ=0.261
all-04
ρ=-0.077
all-05
ρ=0.030
all-06
ρ=0.027
all-07
ρ=-0.062
all-08
ρ=0.181
all-09
ρ=-0.056
all-10
ρ=-0.059
all-11
ρ=0.847
all-12
ρ=0.046

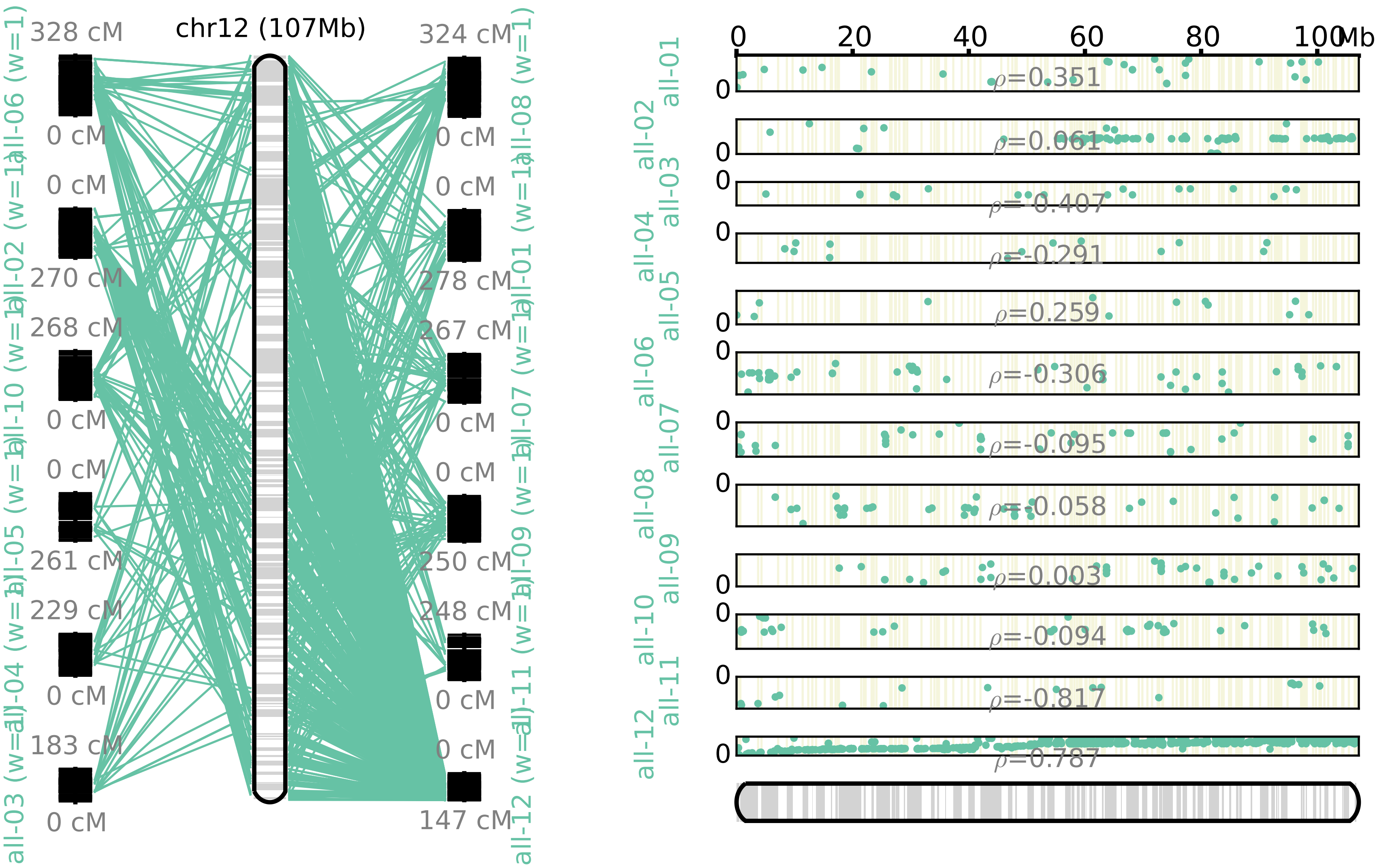

chr12 (107Mb)
328 cM
0 cM
270 cM
268 cM
0 cM
0 cM
261 cM
229 cM
0 cM
183 cM
0 cM
324 cM
0 cM
278 cM
267 cM
0 cM
0 cM
250 cM
248 cM
0 cM
0 cM
147 cM
all-06 (w=1)
all-02 (w=1)
all-10 (w=1)
all-05 (w=1)
all-04 (w=1)
all-03 (w=1)
all-08 (w=1)
all-01 (w=1)
all-07 (w=1)
all-09 (w=1)
all-11 (w=1)
all-12 (w=1)
0
20
40
60
80
100
Mb
all-01
ρ=0.351
all-02
ρ=0.061
all-03
ρ=-0.407
all-04
ρ=-0.291
all-05
ρ=0.259
all-06
ρ=-0.306
all-07
ρ=-0.095
all-08
ρ=-0.058
all-09
ρ=0.003
all-10
ρ=-0.094
all-11
ρ=-0.817
all-12
ρ=0.787
