# Supplementary material for: A consensus and saturated genetic map provides insight into genome anchoring, synteny of Solanaceae and leaf- and fruit-related QTLs in wolfberry (Lycium Linn.)
Source: BMC Plant Biol. 2021 Jul 24;21:350. doi: 10.1186/s12870-021-03115-1 (PMC8306383; doi:10.1186/s12870-021-03115-1)
Supplement: Supplementary file 1 — Additional file 1: Figure 1. Two genetic map. (A) The high-density genetic map by resequencing; (B) The consensus genetic map. [file 12870_2021_3115_MOESM1_ESM.pdf]

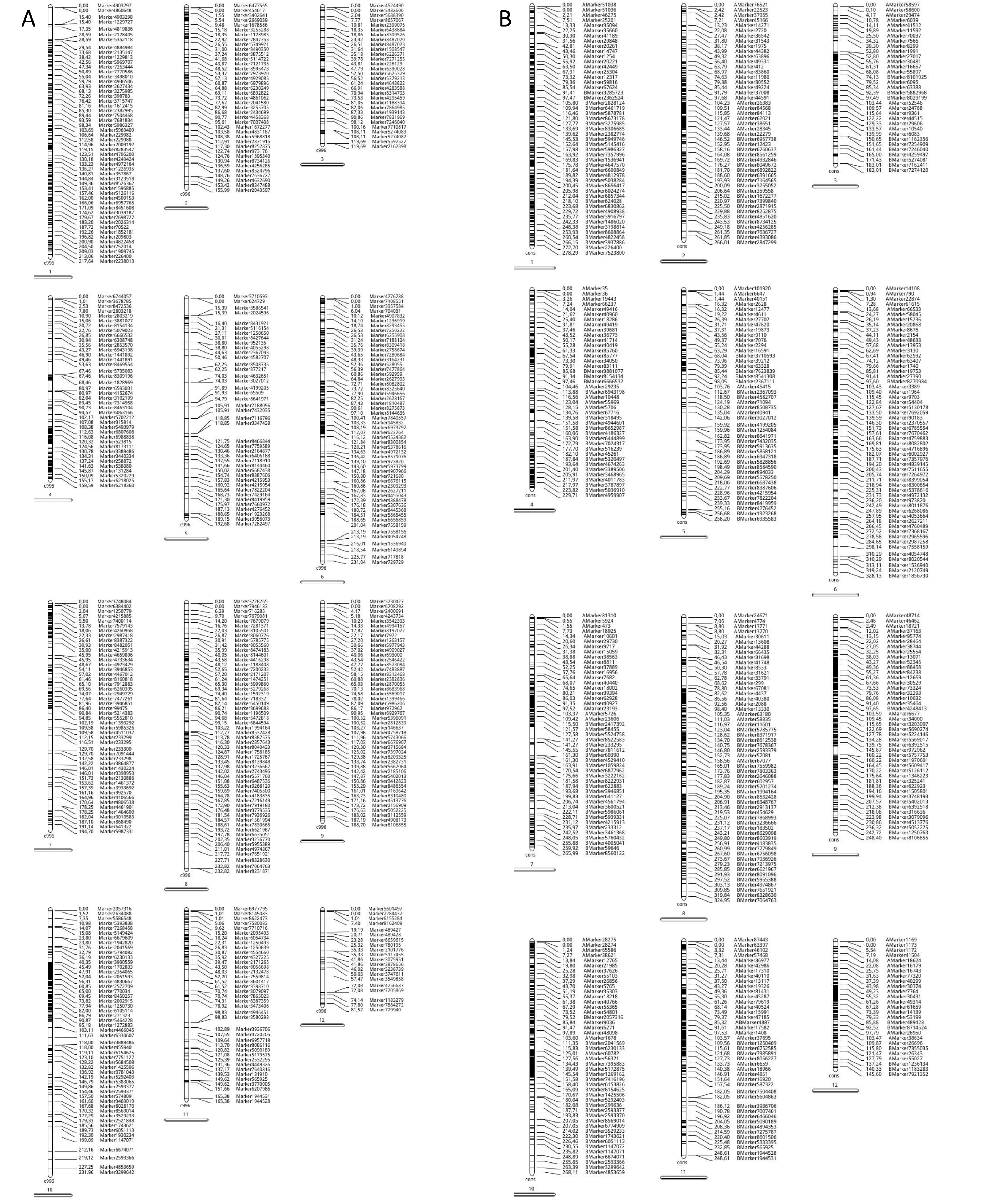
A
B
c996
cons
1
2
3
4
5
6
7
8
9
10
11
12
